# Heterometallic Molecular Architectures Based on Fluorinated β-Diketone Ligands

**DOI:** 10.3390/molecules27227894

**Published:** 2022-11-15

**Authors:** Viktor I. Saloutin, Yulia O. Edilova, Yulia S. Kudyakova, Yanina V. Burgart, Denis N. Bazhin

**Affiliations:** 1 Postovsky Institute of Organic Synthesis, The Ural Branch of the Russian Academy of Sciences, Ekaterinburg 620108, Russia; 2 Department of Organic and Biomolecular Chemistry, Ural Federal University Named after the First President of Russia B.N. Yeltsin, Ekaterinburg 620002, Russia

**Keywords:** fluorinated 1,3-diketones, heterometallic complexes, coordination chemistry, luminescence, transition metals, lanthanides

## Abstract

This review summarizes the data on the synthesis of coordination compounds containing two or more different metal ions based on fluorinated β-diketonates. Heterometallic systems are of high interest in terms of their potential use in catalysis, medicine and diagnostics, as well as in the development of effective sensor devices and functional materials. Having a rich history in coordination chemistry, fluorinated β-diketones are well-known ligands generating a wide variety of heterometallic complexes. In this context, we focused on both the synthetic approaches to β-dicarbonyl ligands with additional coordination centers and their possible transformations in complexation reactions. The review describes bi- and polynuclear structures in which β-diketones are the key building blocks in the formation of a heterometallic framework, including the examples of both homo- and heteroleptic complexes.

## 1. Introduction

Combining different metals in coordination structures and inorganic matrices is a concept of high research interest. Its driving force originates not only from the attractive diversity of molecular structures, but mainly from their exceptional physicochemical properties. Organic ligands are crucial for the successful formation of heterometallic coordination complexes with a discrete or polymeric structure [1,2,3,4,5,6,7]. The possible applications of these compounds are widely investigated in catalytic processes [3,5] and in the search for new luminescent and magnetic materials [4,6], sensors [2,3,7], biologically active agents [1,7], etc.

Being able to form complexes with most of the elements of the periodic table, β-diketones occupy an important place among organic ligands [8,9,10,11,12,13,14,15,16,17,18,19,20,21,22,23,24,25,26]. Moreover, varying substituents at the β-dicarbonyl fragment is an effective tool for the fine-tuning of the physicochemical properties of coordination compounds. In particular, the introduction of one or two fluorinated substituents decreases the intermolecular interactions, thereby reducing the sublimation temperature of the complexes. Along with their thermal stability, these compounds are in high demand in the search for precursors for chemical deposition processes [27]. However, the prevalence of trifluoromethyl β-diketonates compared with non-fluorinated analogues is due to the increased solubility of their complexes and, therefore, the better crystallization [27].

Several reviews have reported the use of β-diketonates as precursors for MOCVD [27,28]. Examples of metal-containing molecular architectures incorporating β-diketonate motifs are described as luminescent materials [29,30]. The achievements in the field of supramolecular metal-containing structures based on poly-β-diketonate ligands are reviewed regularly [9,21,24,25].

This review aims to consider the fluorinated β-diketonates and their involvement in the synthesis of heterometallic structures. Such systematization will reveal the main advantages of the β-diketonates’ chemistry and demonstrate the features of polynuclear systems’ formation and their physicochemical properties.

## 2. Overview of Fluorinated β-Diketones Used as Ligands

**Figure 1:**
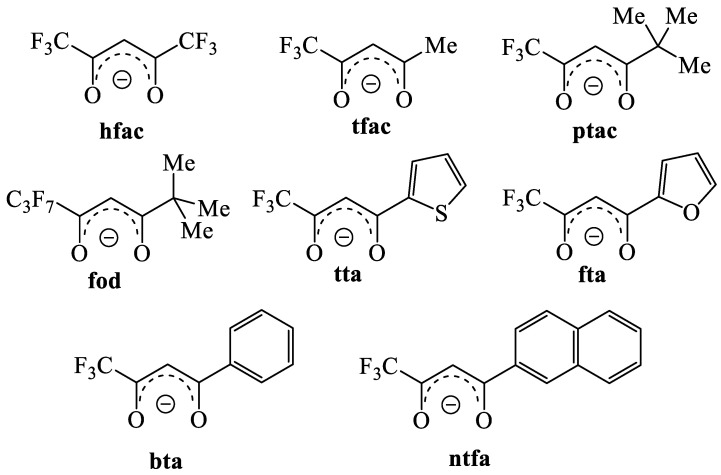
Structures of fluorinated β-diketonate ligands.

The most common fluorinated β-diketones are illustrated in Figure 1. In this series, hfac is the most used ligand: it represents the acac derivative in which the methyl groups are replaced by trifluoromethyl substituents. Containing trifluoromethyl and thiophene substituents, tta is a preferred coligand in the design of luminescent and magnetic complexes. The trifluoromethyl group predominates among the other β-dicarbonyl backbone substituents in the structures of the known fluorinated β-diketonates involved in the synthesis of homo- and heteroleptic complexes.

## 3. The Synthesis of Fluorinated Ligands, Containing the β-Dicarbonyl Fragment

**Scheme 1:**
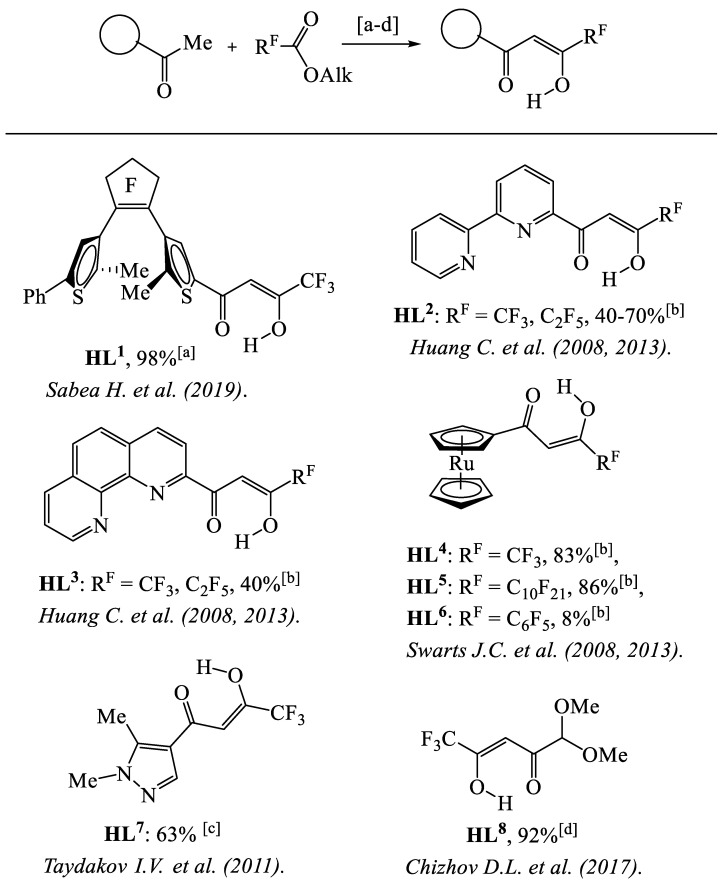
The synthesis of fluorinated functionalized β-diketones [51,52,53,54,55,56,57]. Reaction conditions: [a] LDA, THF, −78 °C, then HCl_aq_; [b] Na, benzene, reflux; [c] NaH, THF, EtOH (cat.), then AcOH; [d] CaH_2_, MeOH, then Cu(OAc)_2_ followed by the decomposition with Na2EDTA_aq_.

In this part, we show the approaches to the original fluorinated β-diketones, which were further used in the synthesis of heterometallic complexes. The Claisen condensation is known as the main synthetic method for the preparation of fluorinated β-diketones. The most frequently used condensing agents are NaH [31,32,33,34,35] and MeONa [36,37,38,39,40,41,42,43], while LiH [44,45,46,47,48], LDA [49] and Na [50] are less demanded. Ethers (DME, Et_2_O, THF) are more preferred solvents rather than benzene or alcohols. The synthesis of functionalized ligands incorporating the β-dicarbonyl fragment and fluorinated groups is shown in Scheme 1 [51,52,53,54,55,56,57]. In this case, the condensation proceeds between the fluorine-containing esters and methyl ketones bearing an aryl, hetaryl substituent or a functional group.

**Scheme 2:**
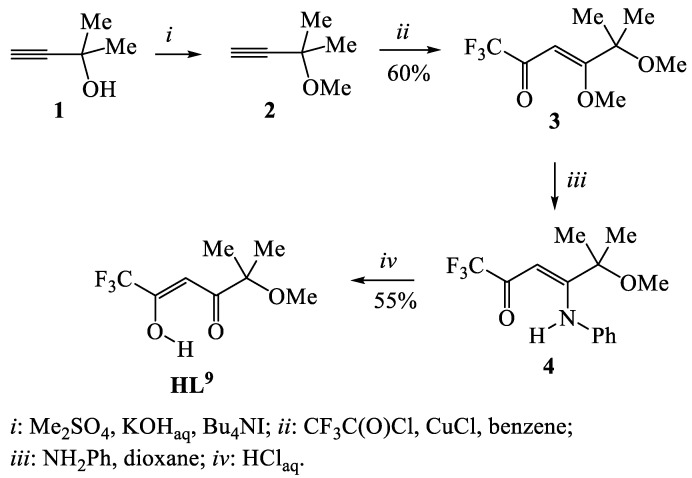
The synthesis of methoxy-substituted CF_3_-β-diketone.

Trifluoromethylated β-diketone (**HL^9^**) with a bulky substituent bearing a methoxy group was obtained as a result of a multi-stage synthesis (Scheme 2) [58]. Alkylation with dimethyl-substituted propargyl alcohol (**1**) resulted in acetylene (**2**) containing a methoxy group at a tertiary carbon atom. Further trifluoroacetylation of unsaturated ether (**2**) gave alkoxyenone (**3**) that reacted with aniline to form the corresponding enaminoketone (**4**). At the last stage, an asymmetric diketone **HL^9^** was isolated under acid hydrolysis.

**Scheme 3:**
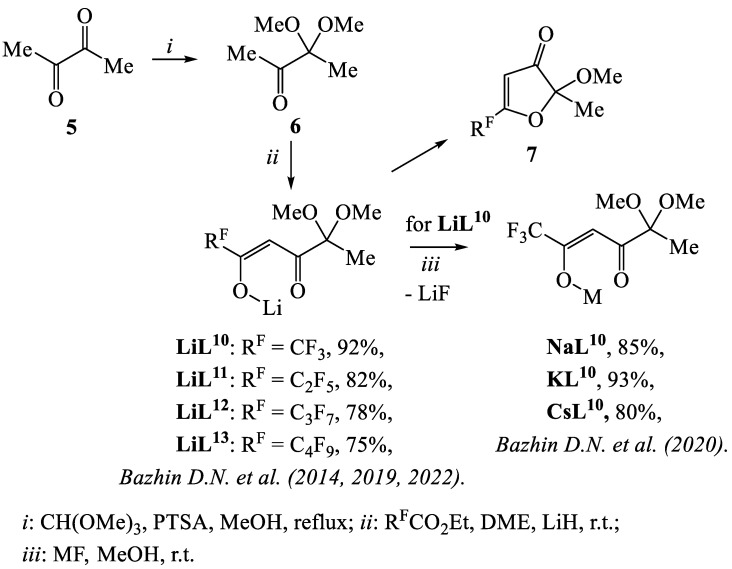
The synthesis of fluorinated acetal-containing β-diketonates [59,60,61,62].

Bazhin et al. later developed a more convenient approach to the synthesis of the methoxy-substituted β-diketone analogue [59]. For this purpose, a commercially available 2,3-butanedione (**5**) was used, in which one of the carbonyl groups can be easily transformed into an acetal fragment (Scheme 3). Further Claisen condensation of the obtained functional ketone (**6**) with fluorinated esters gave the acetal-containing lithium β-diketonates **LiL** [60,61]. The resulting lithium derivatives are easy to isolate in a pure form; they are stable and can be stored under normal conditions. However, these lithium salts were readily converted into 5-(perfluoroalkyl)furan-3(2*H*)-ones (**7**) under weakly acidic conditions and in the presence of the strong Lewis acids (e.g., BF_3_·Et_2_O) [45,46,59]. On the other hand, the direct coordination of lithium β-diketonates with metal ions did not require any bases. Moreover, an efficient method for replacing lithium with another alkali metal ion by the action of the corresponding fluoride was proposed (Scheme 3) [62].

**Scheme 4:**
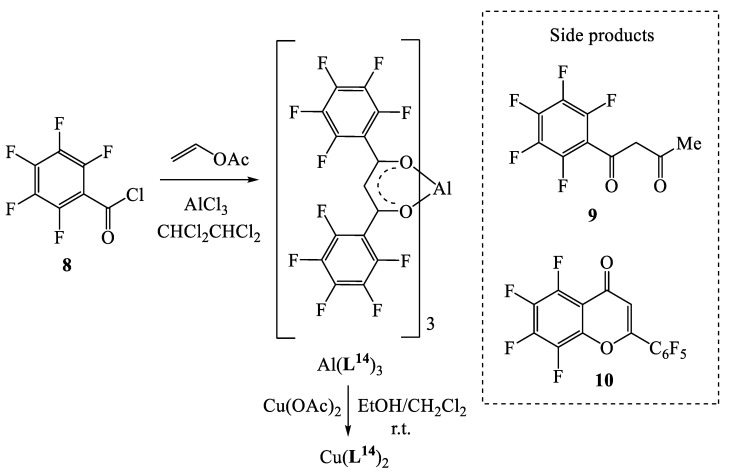
Synthesis of C_6_F_5_-substituted β-diketone.

To synthesize a C_6_F_5_-substituted β-diketone, the reaction between pentafluorobenzoyl chloride (**8**) and vinyl acetate was carried out in the presence of aluminum(III) chloride (Scheme 4) [63,64]. The condensation was accompanied by side products: an asymmetric β-diketone (**9**) with one perfluorophenyl substituent and a fluorinated chromone (**10**) derived from intramolecular cyclization. Aluminum(III) β-diketonate Al(**L^14^**)_3_ easily reacted with divalent transition metal ions (copper, cobalt, nickel) [63], thereby avoiding the isolation of the corresponding bis(pentafluorobenzoyl)methane (**HL^14^**) unstable in a free form.

The above analysis showed the structures of fluorinated 1,3-diketones used as ligands in the synthesis of organometallic derivatives are limited. In this context, functionalized 1,3-dicarbonyl compounds are the least studied, although they have much potential for the formation of heterometallic complexes.

## 4. Coordination Modes of Fluorinated β-Diketonates

**Figure 2:**
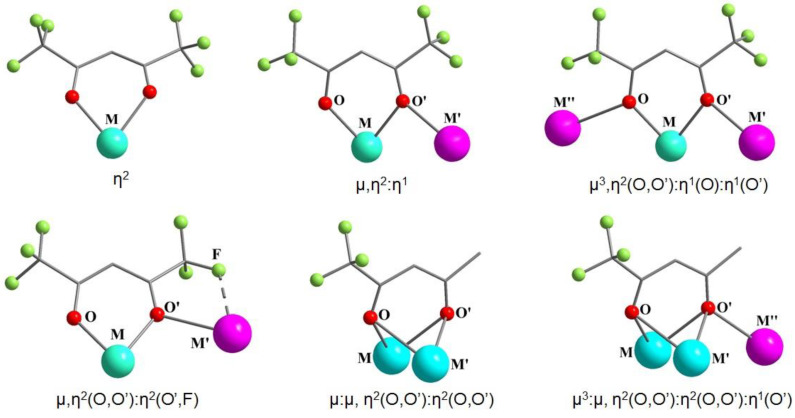
Possible coordination modes of fluorinated β-diketones.

The variety of coordination structures based on β-diketones arises from their ability to act as bi- and polydentate ligands, depending on the metal ions and coligands used [27]. Moreover, the fluorine atoms of the substituent in the β-dicarbonyl framework can participate in the extra coordination with metal ions. In this case, the interatomic distance M…F is less than the sum of the van der Waals radii of fluorine and metal ions indicating non-covalent interaction between them. The principal coordination modes of fluorinated β-diketonates are shown in Figure 2.

## 5. Heterometallic Complexes Containing Bridging β-Diketonate Anions

**Scheme 5:**
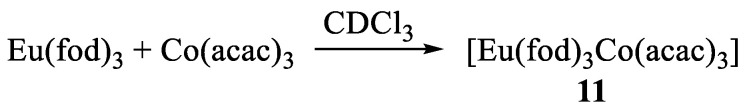
Synthesis of the heterometallic complex [Eu-Co] (**11**).

**Figure 3:**
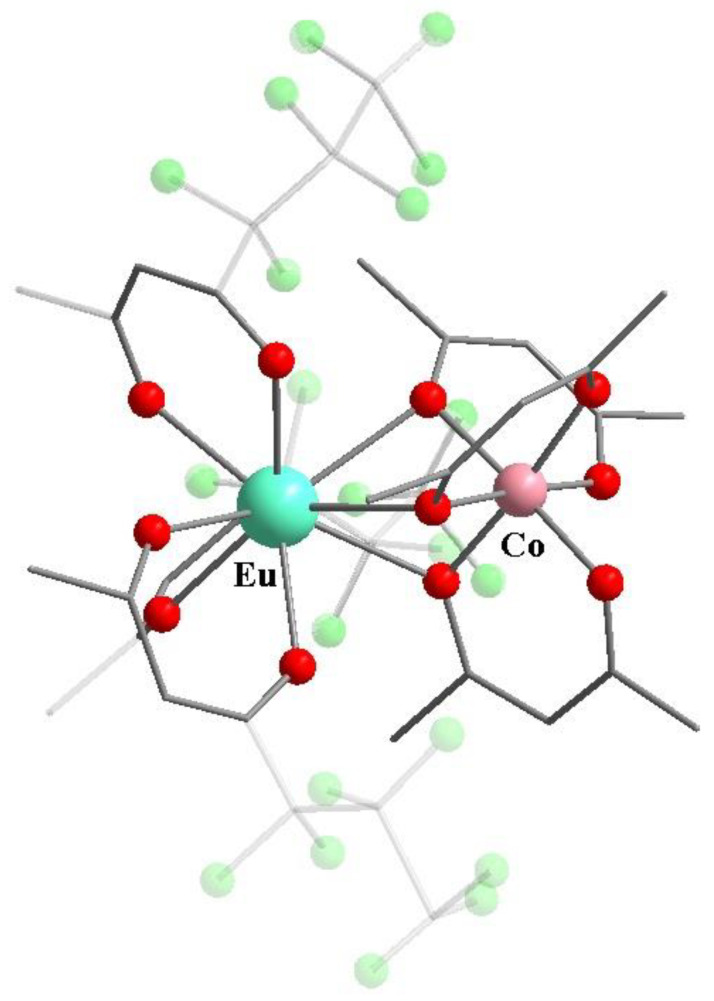
Molecular structure of **11**. Hydrogen atoms and some methyl groups have been omitted, C_3_F_7_ groups of fod are transparent for clarity. The cif has been retrieved from CCDC. CCDC number is 1100888.

The co-crystallization of β-diketonates with various metal ions is known as one of the available methods for the synthesis of heterometallic compounds with both discrete and polymer coordination structures. For example, Lindoy et al. described the heterometallic complex [Eu-Co] (**11**) based on 3d and 4f metal β-diketonates: the stoichiometric structure was formed from two β-diketonates containing europium(III) and cobalt(III) in a CDCl_3_ solution (Scheme 5) [65]. In this case, oxygen atoms of non-fluorinated β-diketonate acted as bridging atoms between two metal ions (Figure 3).

**Scheme 6:**
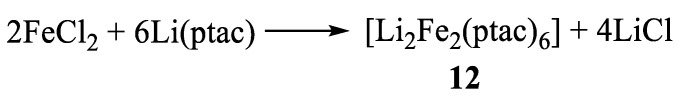
Synthesis of tetranuclear heterometallic complex [Li-Fe] (**12**).

**Figure 4:**
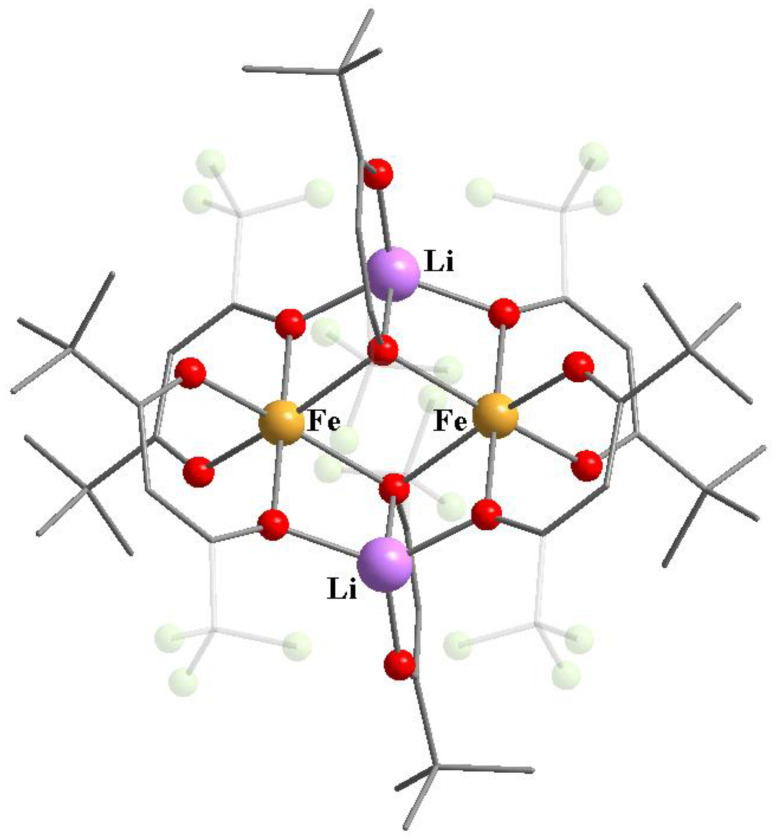
Molecular structure of **12**: (dik = ptac) [66]. Hydrogen atoms have been omitted, CF_3_ groups of ptac are transparent for clarity. The cif has been retrieved from CCDC. CCDC number is 1518385.

The reaction of the iron(II) chloride and the fluorinated lithium β-diketonate led to a tetranuclear heterometallic complex [Li-Fe(II)] (**12**) (Scheme 6) [66]. The resulting iron(II) bis-β-diketonates were coordinated with two molecules of the initial lithium β-diketonate (Figure 4). The coordination environment of metal centers [LiO_5_] and [FeO_6_] consisted only of the oxygen atoms of β-diketonate anions without involving the fluorine atoms.

**Scheme 7:**
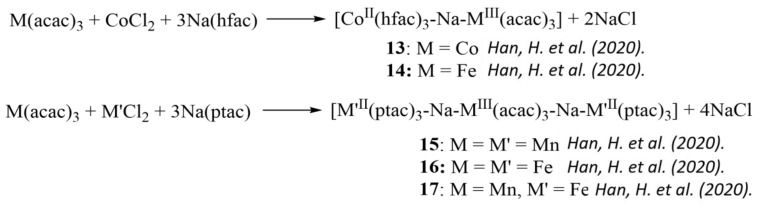
Synthesis of polynuclear M(II)/M(III) β-diketonates [67,68].

**Figure 5:**
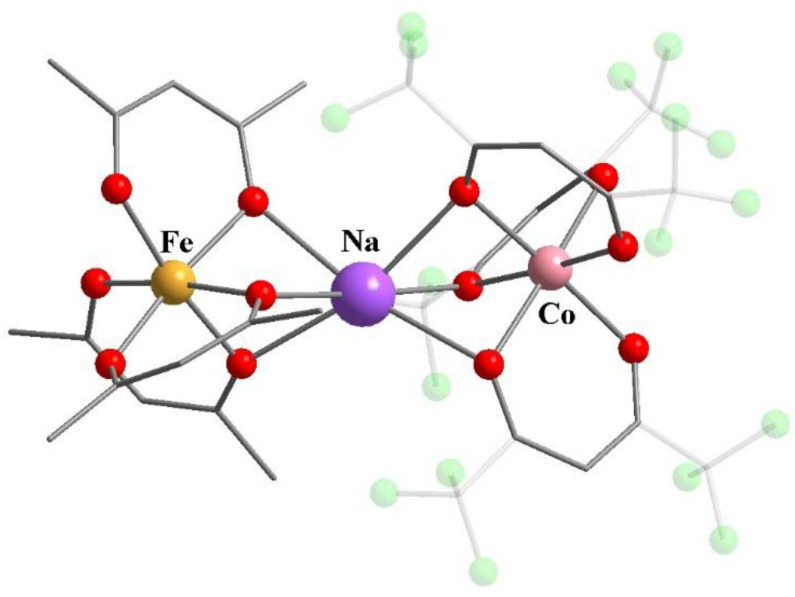
Molecular structure of **13** [67]. Hydrogen atoms have been omitted, CF_3_ groups of hfac are transparent for clarity. The cif has been retrieved from CCDC. CCDC number is 1964370.

**Figure 6:**
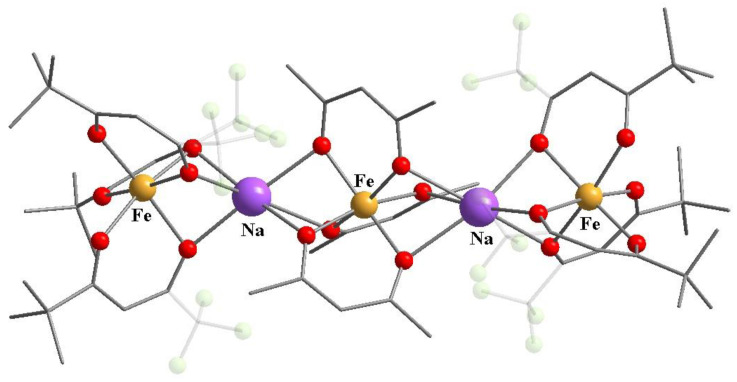
Molecular structure of **16** [68]. Hydrogen atoms have been omitted, CF_3_ groups of hfac are transparent for clarity. The cif has been retrieved from CCDC. CCDC number is 1998878.

**Figure 7:**
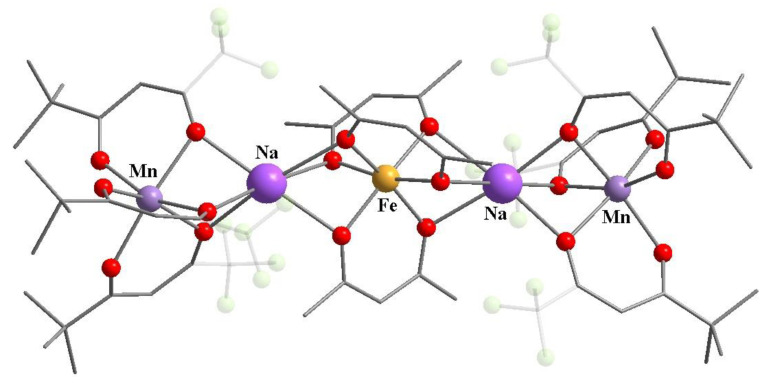
Molecular structure of **17** [68]. Hydrogen atoms have been omitted, CF_3_ groups of hfac are transparent for clarity. The cif has been retrieved from CCDC. CCDC number is 1998879.

In the synthesis of polynuclear heterometallic complexes **13–17,** alkali metal ions act as binders between two fragments of transition metal β-diketonates [67,68]. The initial reagents for these transformations are transition metals(III) β-diketonates based on acac, fluorine-containing sodium β-diketonates and chlorides of divalent 3d metal ions (Scheme 7, Figure 5, Figure 6 and Figure 7). The replacement of one CF_3_ group in the fluorinated ligand with a sterically bulky *t*-butyl group enables the number of metal atoms to be increased from three to five in the complex (Figure 6 and Figure 7). The assembly of trimetallic complexes **13**, **14** was based on the transition metal ions of different oxidation states. In this case, the metal(III) ion was coordinated to non-fluorinated β-diketonate, which was not exchanged for hfac during the reaction.

**Scheme 8:**
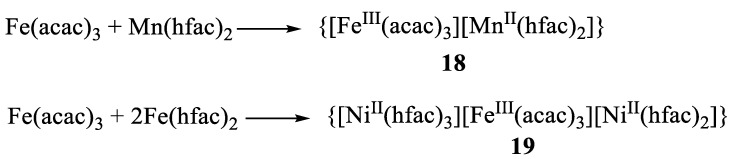
Synthesis of polynuclear M(II)/Fe(III) β-diketonates.

**Figure 8:**
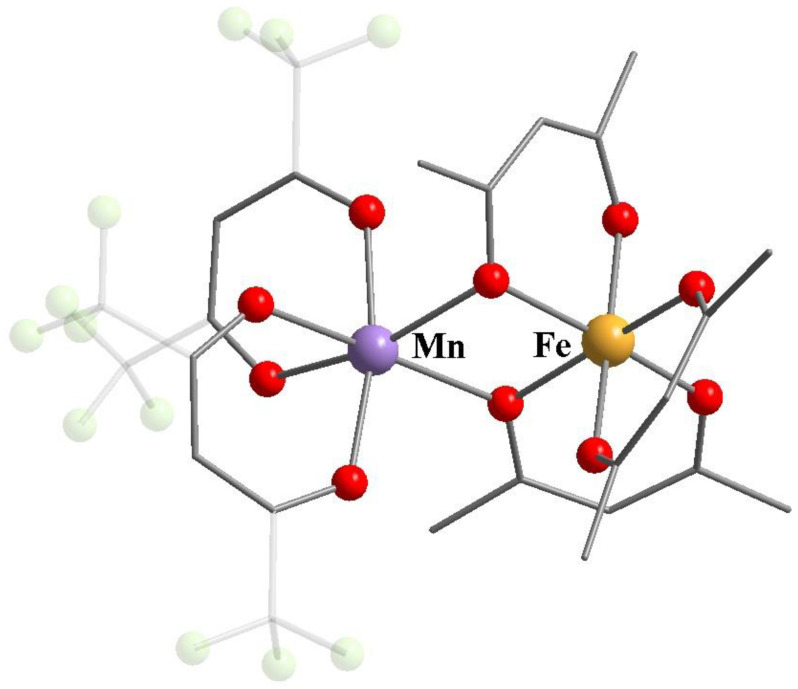
Molecular structure of **18** [69]. Hydrogen atoms have been omitted, CF_3_ groups of hfac are transparent for clarity. The cif has been retrieved from CCDC. CCDC number is 1040552.

**Figure 9:**
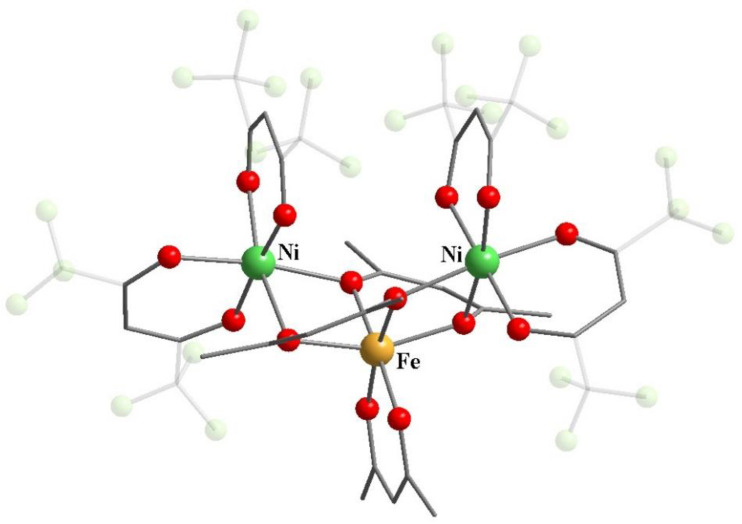
Molecular structure of **19** [69]. Hydrogen atoms have been omitted, CF_3_ groups of hfac are transparent for clarity. The cif has been retrieved from CCDC. CCDC number is 1040553.

The co-crystallization of two 3d β-diketonates with metals in different oxidation states resulted in [Fe^III^(acac)_3_][Mn^II^(hfac)_2_] (**18**) and [Ni^II^(hfac)_2_][Fe^III^(acac)_3_][Ni^II^(hfac)_2_] (**19**) (Scheme 8) [69]. Similarly to heterometallic complexes **13**–**17**, fluorinated β-diketonate was coordinated to a metal ion with the lowest oxidation number. In the structures of **18**, **19** the oxygens of non-fluorinated β-diketonate were bridging atoms, which filled the coordination sphere of Mn(II) or Ni(II) ions coordinating the hfac (Figure 8 and Figure 9). Authors have postulated that the transition metal(II) center participating in bridging interactions with oxygen atoms of neighboring unit(s) should be highly Lewis acidic because of the chelation by diketonates with electron-withdrawing groups. In contrast, the transition metal(III) counterpart should have sterically uncongested ligands with electron-donating substituents provoking diketonate oxygen atoms’ involvement in bridging interactions with the M(II) center.

**Scheme 9:**
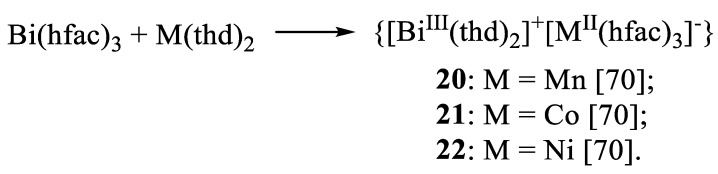
Synthesis of heteroleptic [Bi(III)-M(II)] complexes (**20**–**22**).

**Figure 10:**
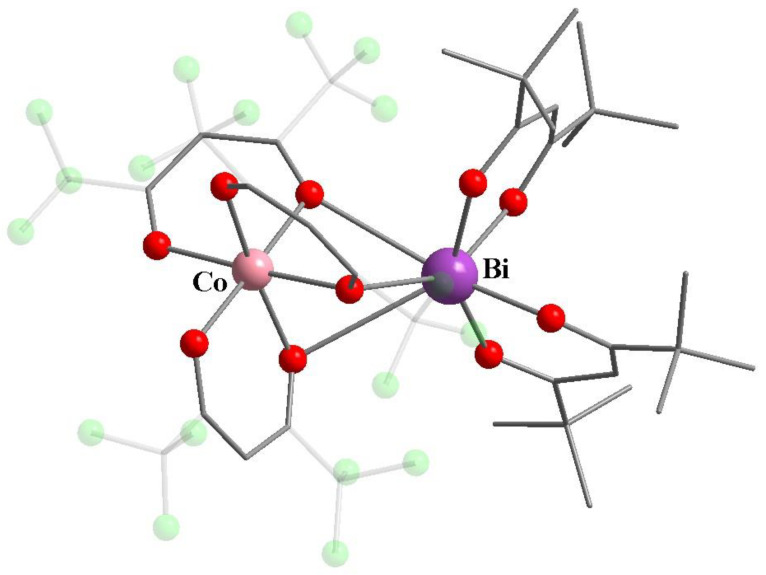
Molecular structure of **21** [70]. Hydrogen atoms have been omitted, CF_3_ groups of hfac are transparent for clarity. The cif has been retrieved from CCDC. CCDC number is 1475305.

The solid-state reaction of precursors containing metals of different oxidation states led to heteroleptic [Bi(III)-M(II)] (**20**–**22**) complexes, where M(II) was a 3d transition metal ion (Scheme 9) [70]. In this case, the bridging oxygen atoms between metal centers were from the hfac ligands of the anionic *tris*-diketonate fragment (Figure 10).

**Scheme 10:**
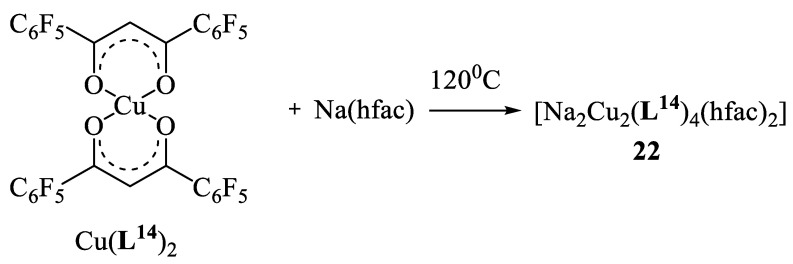
Synthesis of heterometallic [Na-Cu] complex (**22**).

**Figure 11:**
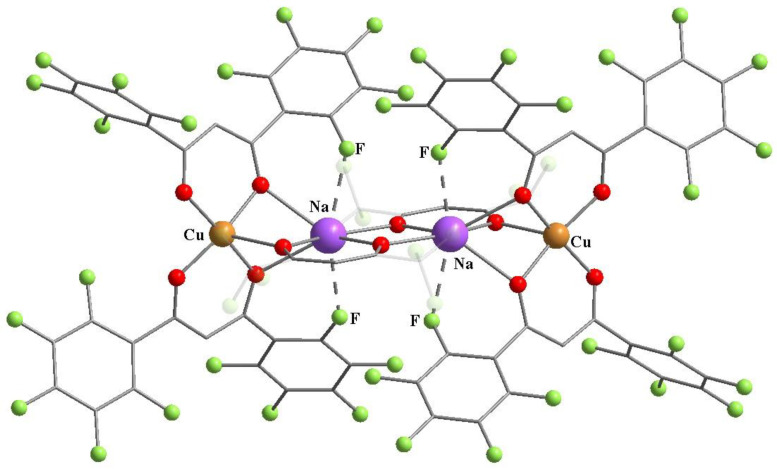
Molecular structure of **22** [64]. Hydrogen atoms have been omitted, CF_3_ groups of hfac are transparent for clarity. The cif has been retrieved from CCDC. CCDC number is 1850164.

Dikarev et al. described the first example of the heterometallic [Na-Cu] complex (**22**) resulting from two different fluorinated β-diketonates [64]. The reaction of unsolvated [Cu(**L^14^**)] and Na(hfac) gave a heterometallic β-diketonate [Na_2_Cu_2_(pfbm)_4_(hfac)_2_] (**22**) (Scheme 10). The discrete structure of the solvent-free [Na-Cu] environment consisted of a dimeric [Na_2_(hfac)_2_] unit surrounded by two Cu(pfbm)_2_ fragments (Figure 11). β-diketonates adopted the chelating-bridging mode between Cu and Na by coordinating through one oxygen atom in the case of F-aryl-containing ligands and two oxygen atoms in the case of hfac anions. The overall distorted square antiprismatic coordination environment for Na was formed by the five primary Na–O interactions (av. 2.41 Å) and three secondary Na–F contacts (av. 2.60 Å).

**Figure 12:**
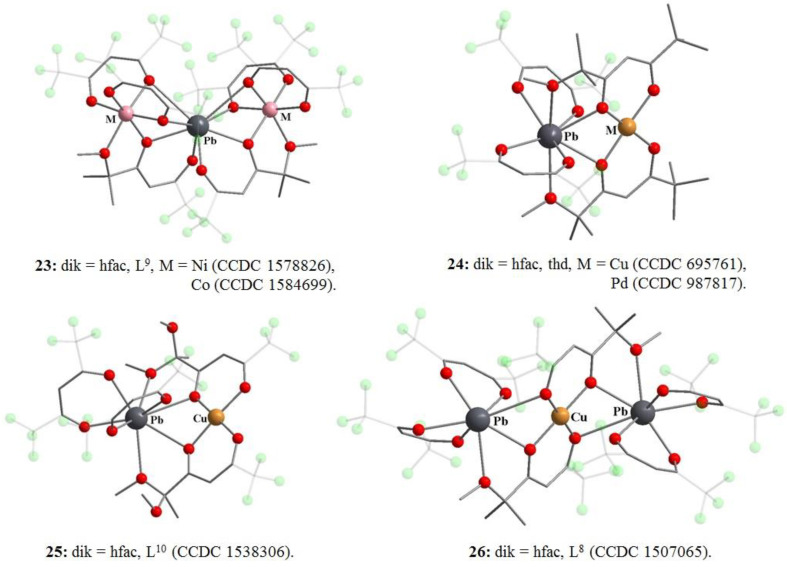
Molecular structures of **23**–**26** [71,72,73,74]. Hydrogen atoms have been omitted, CF_3_ groups are transparent for clarity. The cifs have been retrieved from CCDC.

The methoxy substituent in thd, **L^9^**, **L^10^**, participated in the additional coordination with metal ions during the formation of polynuclear structures [71,72,73]. The co-crystallization of two different β-diketonates led to the discrete heterometallic complexes, e.g., [Pb-M] (**23**–**26**) (Figure 12). The thd is the unique structure because it contains a sterically bulky group close to the β-dicarbonyl fragment that is a necessary condition for the synthesis of discrete heterometallic complexes. If this does not happen, then coordination [Pb-Cu] polymers are formed in the absence of methoxy substituents at the β-dicarbonyl fragment [73,74]. In polymeric fluorinated [Pb-Cu] structures **23**–**26,** the square planar chelate Cu(dik)_2_ built up an octahedral coordination environment due to the bridging oxygen atoms of the ligands from Pb(hfac)_2_ moieties.

## 6. Lanthanide(III) *tetrakis*-β-Diketonates

**Scheme 11:**
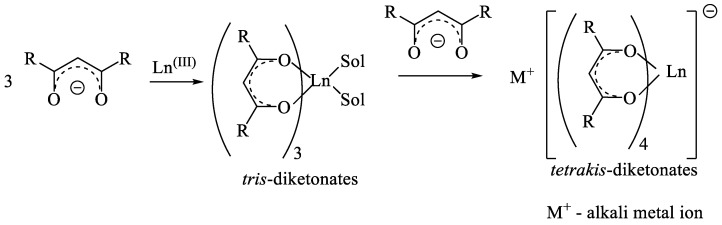
Synthesis of lanthanide(III) *tris*-/*tetrakis*-β-diketonates.

In general, the reaction of β-diketones with lanthanide(III) salts leads to the substitution of halide, carboxylate and nitrate anions of metals with a β-diketonate anion. The coordination of transition trivalent 4f-metals with two or three β-diketonate anions provided the discrete neutral complexes [M(dik)_3_(coligand)_2_] (Scheme 11) [25,29,32,33,39,75,76,77]. However, the metal ion was additionally coordinated to the solvent molecules or bidentate ligands having the donor heteroatoms (oxygen, nitrogen). At the next step, the metal(III) *tetrakis*-β-diketonates containing the anionic part of [M(dik)_3_]^−^ or [M(dik)_4_]^−^,were formed as a result of the β-diketonate anion addition to the central metal atom in neutral *tris*-β-diketonates.

**Figure 13:**
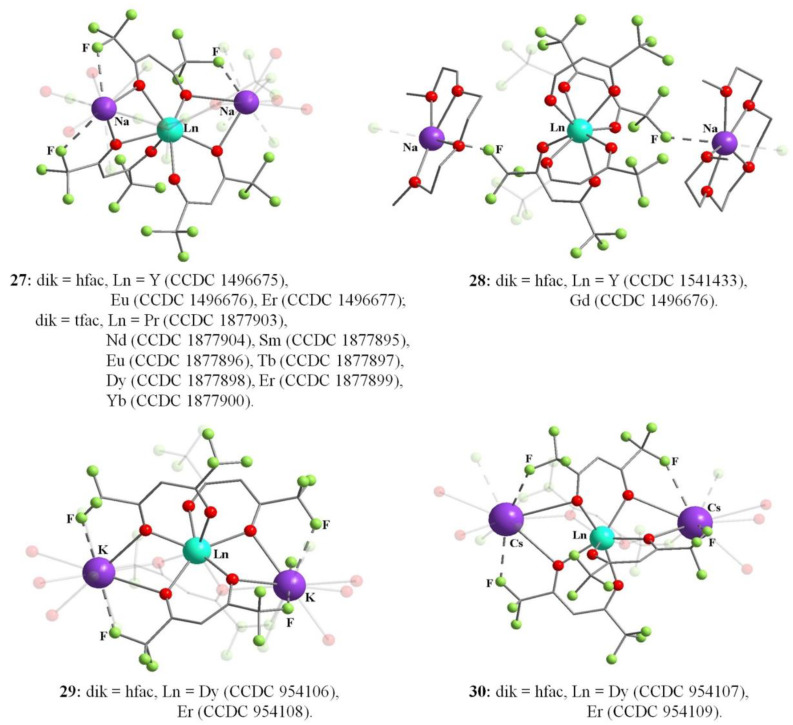
Molecular structures of Ln(III) *tetrakis*-diketonates **27**–**30** [78,79,80,81]. Hydrogen atoms and some CF_3_ groups have been omitted for clarity. The cifs has been retrieved from CCDC.

*Bi*heterometallic *tetrakis*-β-diketonates can form both the discrete or polymer structures depending on the nature of the substituents in β-diketonate and coligands [78,79,80,81,82,83,84,85]. In the polymer chains **27**–**30**, alkali metal ions, including sodium, potassium and cesium, acted as linkers between two adjacent *tetrakis*-β-diketonate fragments (Figure 13) [78,79,80,81]. In this case, both the oxygen and fluorine atoms of the β-diketonate were coordinated to alkali metals. A polyether molecule can be used as a polydentate coligand to fill the coordination environment of the alkali metal ion (**28**) [80]. The complexes Na[Ln(hfac)_4_] (**27**) were found to be highly volatile and stable in the gas phase while preserving the heterometallic fragment. The decay of heterometallic [Ln-Na] complexes (**27**) under argon atmosphere yielded the phase-pure NaLnF_4_ [78]. Varying the decay temperature, the formation of one of the NaLnF_4_ allotropes can be controlled [78]. Another approach was to use two precursors [Na(hfac)(tetraglyme)] and [Ln(hfac)_3_(diglyme)] together in sol–gel synthesis of the hexagonal phase of β-NaYF_4_:Yb^3+^,Er^3+^ at an elevated temperature [84].

**Figure 14:**
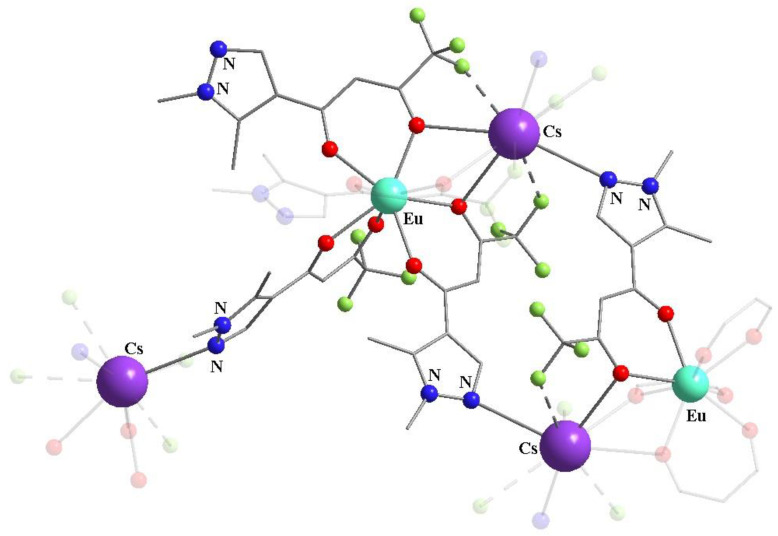
Structure X-ray structure of one-dimensional chain in **31** [82]. Hydrogen atoms have been omitted, some diketonate ligands and CF_3_ groups are transparent for clarity. The cif has been retrieved from CCDC. CCDC number is 922987.

Diketone **HL^7^** with a pyrazole substituent formed the crystal packing of the heterometallic [Eu-Cs] complex (**31**) due to the coordination of nitrogen atoms of the heterocyclic system with cesium ions (Figure 14) [82]. The coordination environment of cesium(I) ions is saturated by nitrogen atoms of pyrazole, fluorine atoms of CF_3_ groups and bridging oxygen atoms of diketonate anions. The contact length of Cs…F is ~3.21–3.44 Å, which is comparable with the bond length of Cs…N equal to ~3.17–3.20 Å.

**Scheme 12:**
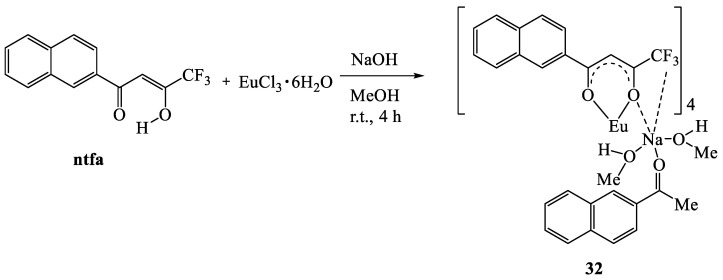
Synthesis of *tetrakis*-β-diketonate [Eu-Na] (**32**).

**Figure 15:**
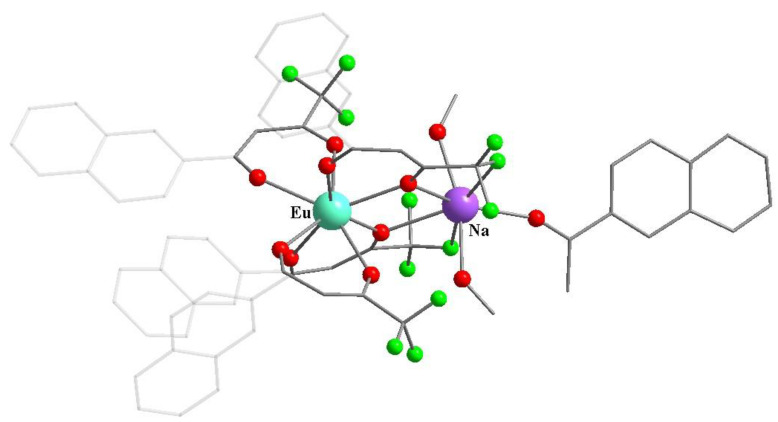
Molecular structure of **32** [83]. Hydrogen atoms have been omitted, some ligands groups are transparent for clarity. The cif has been retrieved from CCDC. CCDC number is 2071833.

The complexation of ntfa with europium(III) chloride in the presence of sodium alkali in methanol gave the discrete [Eu-Na] *tetrakis*-β-diketonate (**32**) containing an acetyl naphthalene (Scheme 12) [83]. The aromatic ketone in the complex **32** indicates a side retro-Claisen reaction involving ntfa. Therefore, coligand contribution led to the change from polymer to discrete *tetrakis*-β-diketonates (Figure 15). Heterometallic complex **32** had good luminescent properties, exhibiting a phosphorescence time equal to 0.595 ms with a quantum yield of 47.5%. Photophysical parameters were measured under excitation at a wavelength of 370 nm in CH_2_Cl_2_ solution [83].

**Figure 16:**
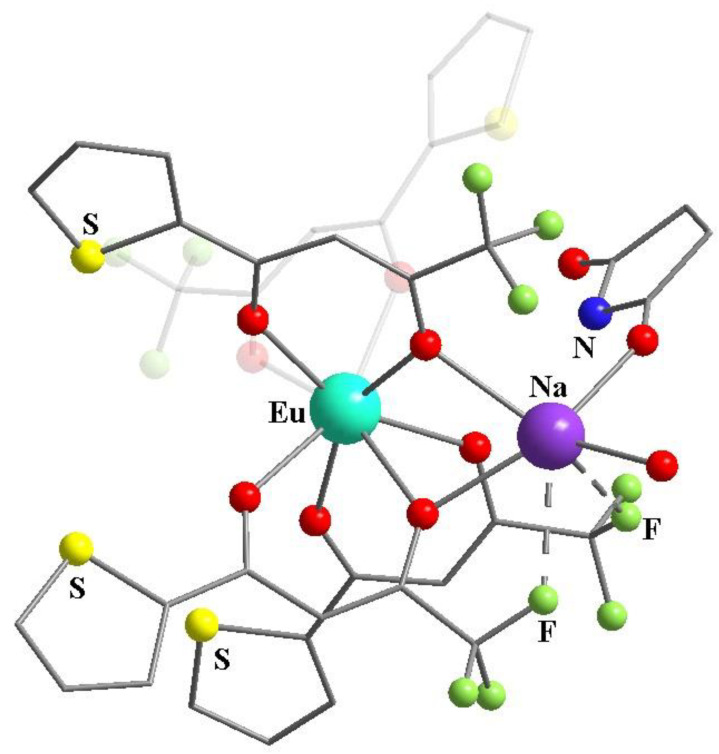
Molecular structure of **33** [85]. Hydrogen atoms have been omitted, some ligand groups are transparent for clarity. The cif has been retrieved from CCDC. CCDC number is 2013280.

Succinimide can replace one of the water molecules from the coordination environment of alkali metal ions, thereby acting as coligand in heterometallic complex **33** (Figure 16) [85]. *Tetrakis*-β-diketonate [Eu-Na] (**33**) based on tta exhibited the high phosphorescence quantum yield of 71% and the afterglow time of 0.84 ms upon excitation at a wavelength of 365 nm.

**Scheme 13:**
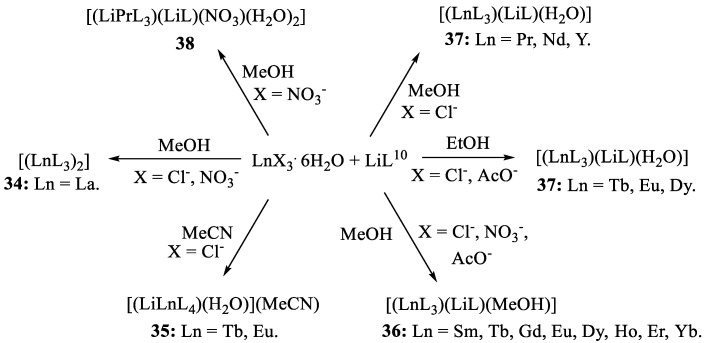
Synthesis of lanthanide(III) complexes based on lithium β-diketonate (**LiL^10^**).

**Figure 17:**
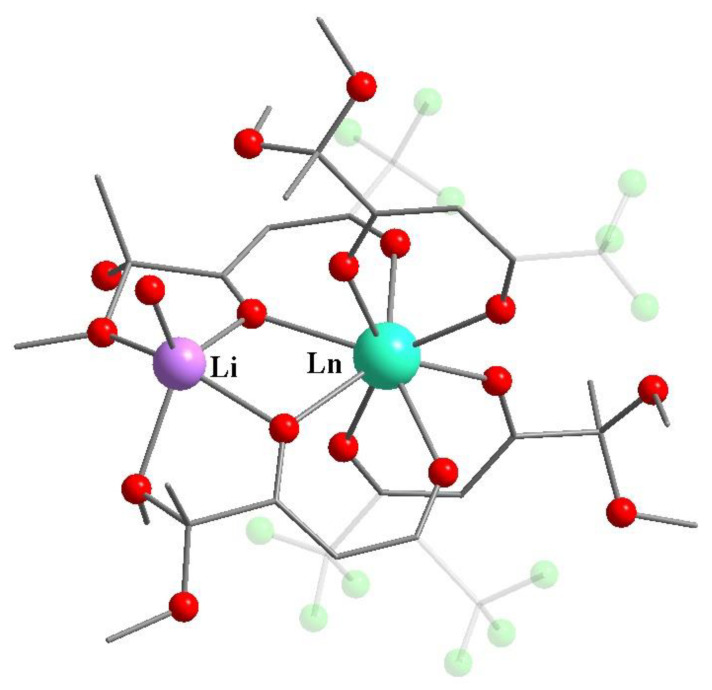
Molecular structure of **35** [87]. Hydrogen atoms, MeCN molecules have been omitted, CF_3_ groups are transparent for clarity. The cif has been retrieved from CCDC. CCDC numbers are 1973632 (Ln = Eu), 1973633 (Ln = Tb).

The functionalized lithium β-diketonate **LiL^10^** reacted with lanthanide(III) ions to form heterometallic compounds of various structures (Scheme 13) [62,86,87,88,89]. An extra functional group in the ligand saturated the metal ion coordination sphere with no additional coligands. Such homoleptic [Ln_2_L_6_] complexes (**34**) were obtained in the case of Ln(III) ions with the largest radius. However, the reaction of lithium diketonate **LiL^10^** with most of the 4f metals led to the discrete β-diketonates **36**–**38** of three types depending on the solvent used (Scheme 13). The first type included “classical” *tetrakis*-β-diketonates **35**, in which four β-diketonate anions were coordinated to lanthanide(III) ion. The methoxy groups of the ligands participate in the additional coordination with the lithium ion, thereby providing the discrete structure of **35** (Figure 17). The [Ln-Li] *tetrakis*-β-diketonates **35** were formed in acetonitrile media and the solvent molecule was also included in the crystal packing. Heterometallic complexes **35** were the first examples of lanthanide-lithium β-diketonates, which have been obtained and characterized by different spectral and crystallographic methods.

**Figure 18:**
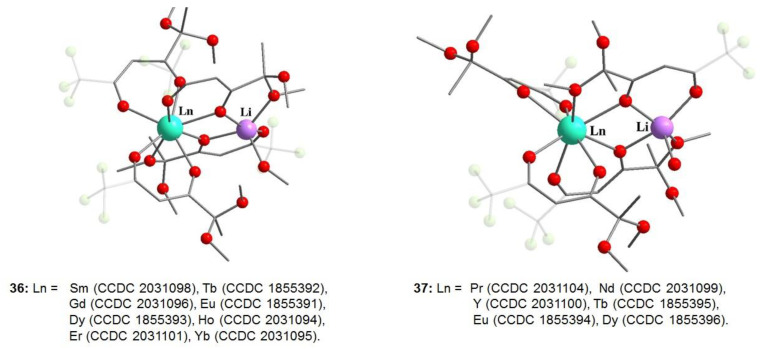
Molecular structures of **36** and **37** (**dik** = **L^10^**) [86,88]. Hydrogen atoms have been omitted, CF_3_ groups are transparent for clarity. The cif has been retrieved from CCDC.

The second and the third types of complexes included the compounds **36**, **37**, in which the Ln(III) *tris*-β-diketonate fragment was coordinated to the initial lithium β-diketonate **LiL^10^** (Scheme 13). The solvent choice (methanol or ethanol) influenced the coligands at the lithium ion in heterometallic complexes **36** (where Ln = Tb, Dy, Eu) (Figure 18). However, the main distinctive characteristic of these close structures was their different crystal packings. Complexes **36** (where Ln = Tb, Dy, Eu) exhibited mechanoluminescent properties, and one of them, [Dy-Li], was found to be a single-molecule magnet with an energy barrier equal to 53 K.

**Figure 19:**
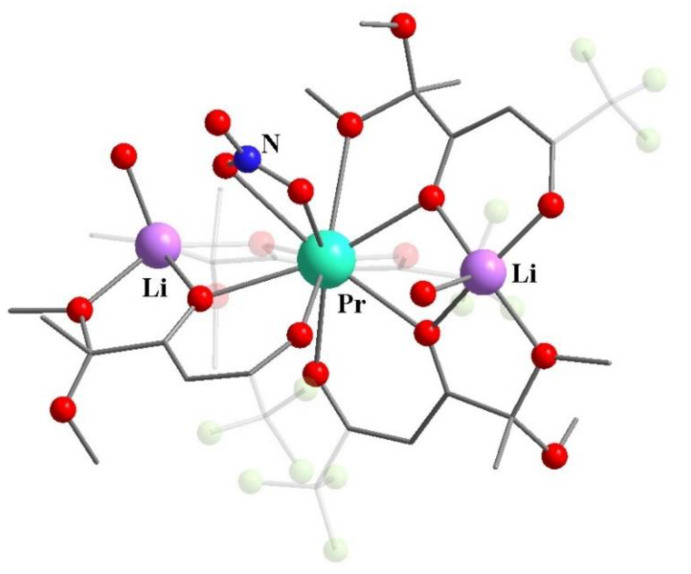
Molecular structure of **38** [86]. Hydrogen atoms have been omitted, CF_3_ groups and one ligand molecule are transparent for clarity. The cif has been retrieved from CCDC. CCDC numbers is 2031103.

**Scheme 14:**
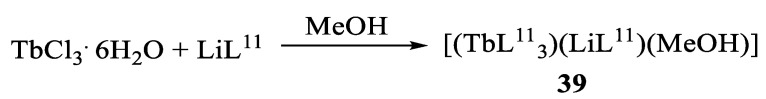
Synthesis of discrete [Tb-Li] diketonate **39**.

The exception was the trimetallic complex **38** formed by **LiL^10^** reaction with praseodymium(III) nitrate (Scheme 14) [86]. Its structure contained a ten-coordinated praseodymium(III) ion and two lithium ions in the coordination with four β-diketonate anions (Figure 19). One of the ligands from the *tris*-β-diketonate fragment was bound with the lithium atom of the initial molecule **LiL^10^**. The coordination environment around the praseodymium(III) ion was saturated by nitrate and three β-diketonate anions, one methoxy group and the bridging oxygen atom of the **LiL^10^** β-dicarbonyl fragment. In addition, an unusual structure of complex **38** included the second lithium ion, which was coordinated to methoxy groups and oxygen atoms from the β-diketonate fragment (Figure 19).

**Figure 20:**
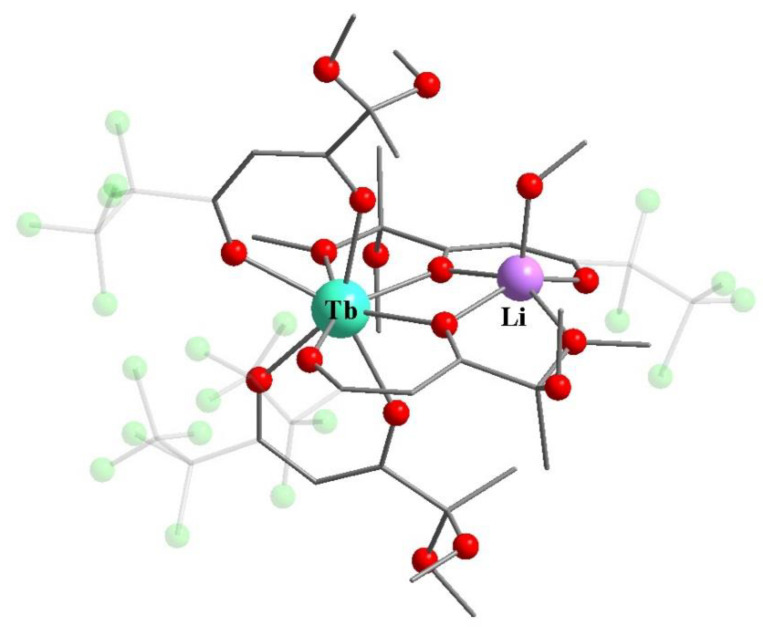
Molecular structure of **39** [89]. Hydrogen atoms have been omitted, C_2_F_5_ groups are transparent for clarity. The cif has been retrieved from CCDC. CCDC numbers is 2011088.

Increasing the length of the fluoroalkyl substituent from CF_3_ to C_2_F_5_ in acetal-containing β-diketonates **LiL^11^** did not change the composition and structure of the resulting heterometallic β-diketonate **39** (Scheme 14, Figure 20) [89]. However, the phosphorescence lifetime decreased for the obtained [Tb-Li] diketonate **39** compared with the trifluoromethyl analog.

**Scheme 15:**
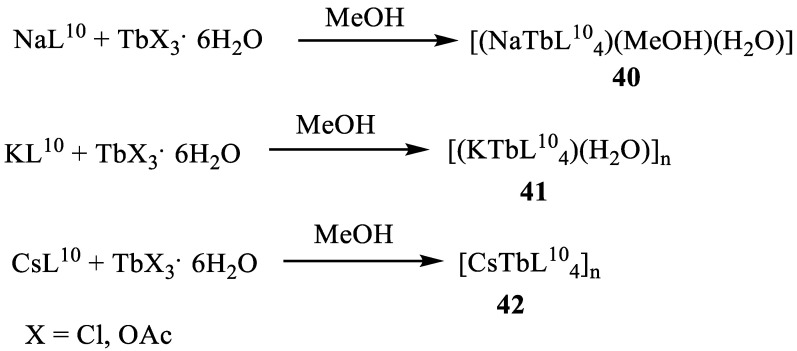
Synthesis of terbium(III) *tetrakis*-β-diketonates **40**–**42**.

**Figure 21:**
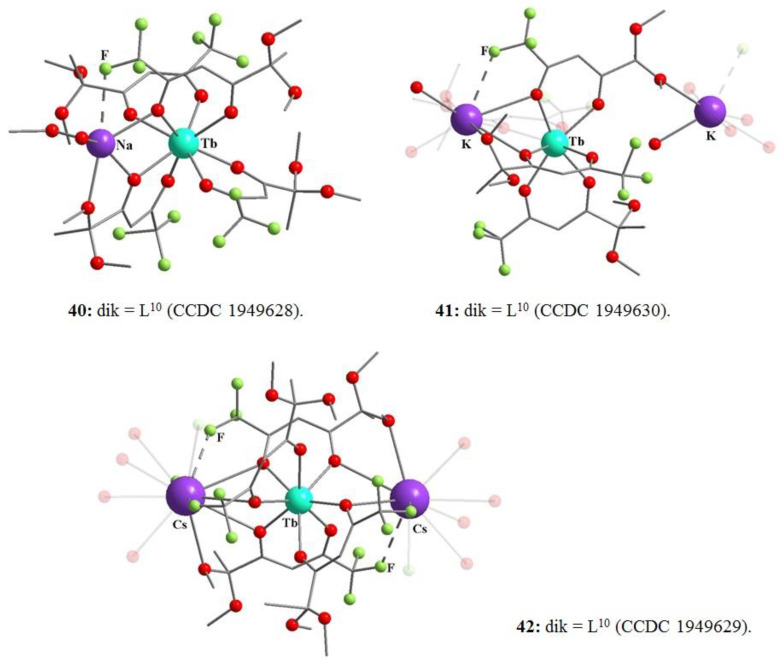
Molecular structures of **40**–**42** [62]. Hydrogen atoms have been omitted, some CF_3_ groups are transparent for clarity. The cif has been retrieved from CCDC.

The acetal-containing β-diketonates of other alkali metals (Na, K, Cs) reacted with terbium(III) chloride to afford the [Tb-M] (M = Na, K, Cs) complexes **40**–**42** (Scheme 15) [62]. In all cases, the lanthanide(III) *tetrakis*-β-diketonates **40**–**42** were formed. Complex [Tb-Na] **40** had a discrete structure, while [Tb-K] **41** and [Tb-Cs] **42** were coordination polymers (Figure 21).

## 7. Heteronuclear Complexes with Isolated Metal Centers

This part describes complexes in which metal centers are not connected by bridging oxygen atoms of both β-diketonates and other coligands.

**Scheme 16:**
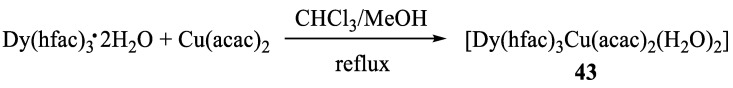
Synthesis of [Dy-Cu] β-diketonate **43**.

**Figure 22:**
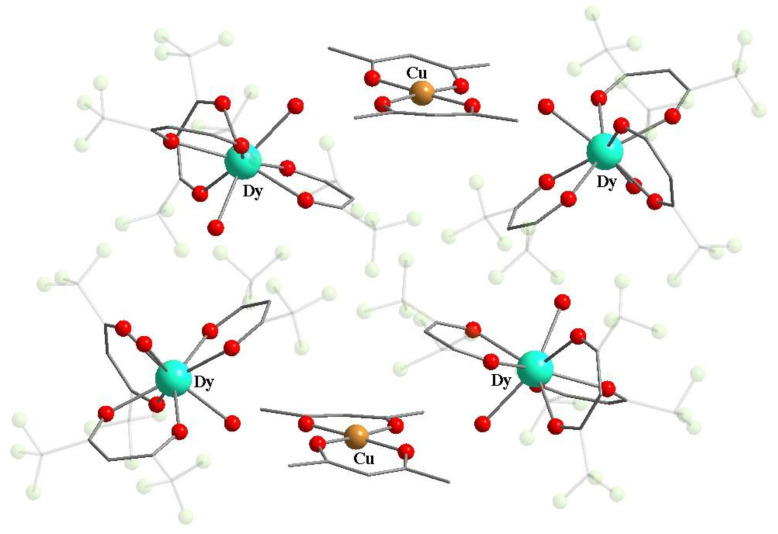
A crystal packing fragment of **43** [90]. Hydrogen atoms have been omitted, some ligand groups are transparent for clarity. The cif has been retrieved from CCDC. CCDC number is 1938913.

The co-crystallization of equimolar amounts of Dy(III) and Cu(II) β-diketonates afforded the heterometallic complex **43** in a good yield (Scheme 16) [90]. Coligands’ exchange around metal ions did not occur in this case (Figure 22). The Cu…Dy had the smallest distance between metal centers in the crystal lattice equal to 5.874 Å. Unlike the original [Dy(hfac)_3_•2H_2_O] complex, a synthesized [Dy-Cu] system (**43**) consisting of mixed β-diketonate ligands exhibited the properties of a molecular magnet with an energy barrier value equal to 55.3 K [90]. 

**Scheme 17:**
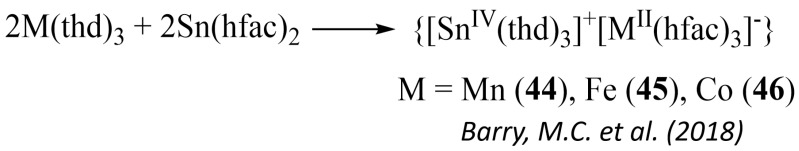
Synthesis of discrete [Sn(IV)-M(II)] complexes (**44–46**) [91].

**Figure 23:**
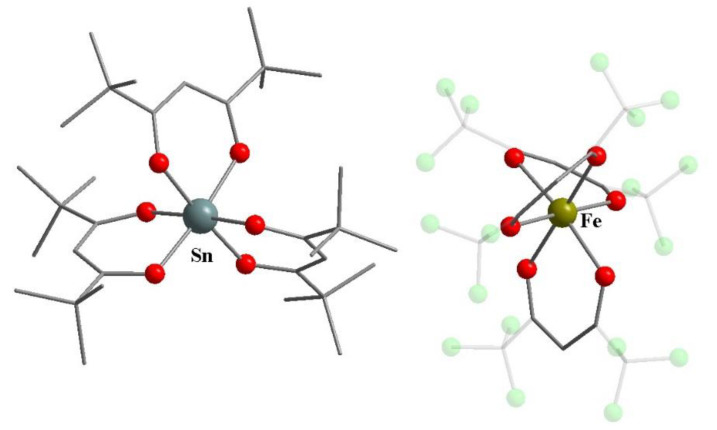
Molecular structure of **45** [91]. Hydrogen atoms have been omitted, CF_3_ groups are transparent for clarity. The cif has been retrieved from CCDC. CCDC numbers is 1811494.

The solid-state redox reaction between tin(II) and 3d metal β-diketonates proceeded with the ligand exchange around the metal centers to give the heterometallic compounds **44**–**46** (Scheme 17) [91]. The obtained bimetallic complexes consisted of two homoleptic *tris*-β-diketonate fragments forming an ion pair (Figure 23).

**Scheme 18:**
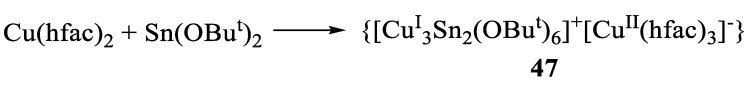
Synthesis of bimetallic [Sn-Cu] complex **47**..

**Figure 24:**
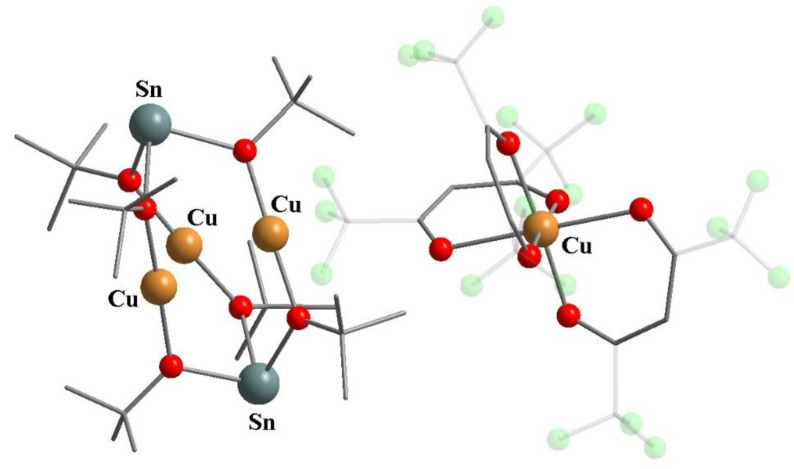
Molecular structure of **47** [92]. Hydrogen atoms have been omitted, some ligand groups are transparent for clarity. The cif has been retrieved from CCDC. CCDC numbers is 1056284.

Copper(II) β-diketonate reacted with tin(II) alkoxide to give the six-nuclear [Sn-Cu] complex (**47**) (Scheme 18) [92]. Similarly to bimetallic [Sn-M] complexes **44**–**46**, an ion pair was formed as a result of the redox reaction. Copper(II) *tris*-β-diketonate represented the anionic part, while the oxygen atoms of tin(II) alkoxides were coordinated to copper(I) ions in the cationic part (Figure 24).

## 8. Binuclear Complexes Based on β-Diketones with an Additional Chelating Cavity

**Scheme 19:**
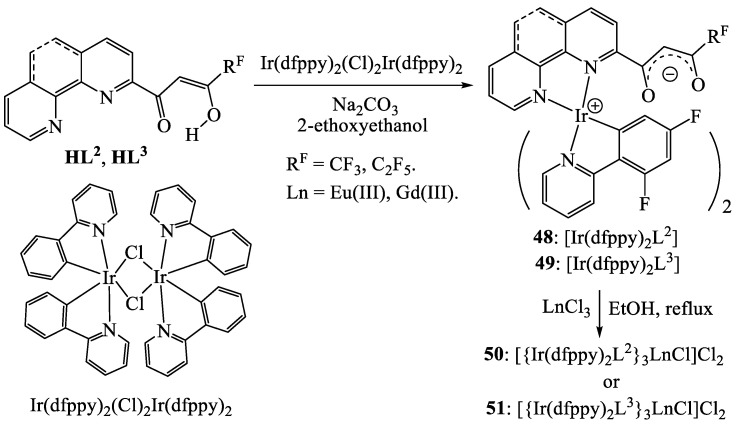
Synthesis of bimetallic [Ir-Eu] complexes **48**, **49**.

**Figure 25:**
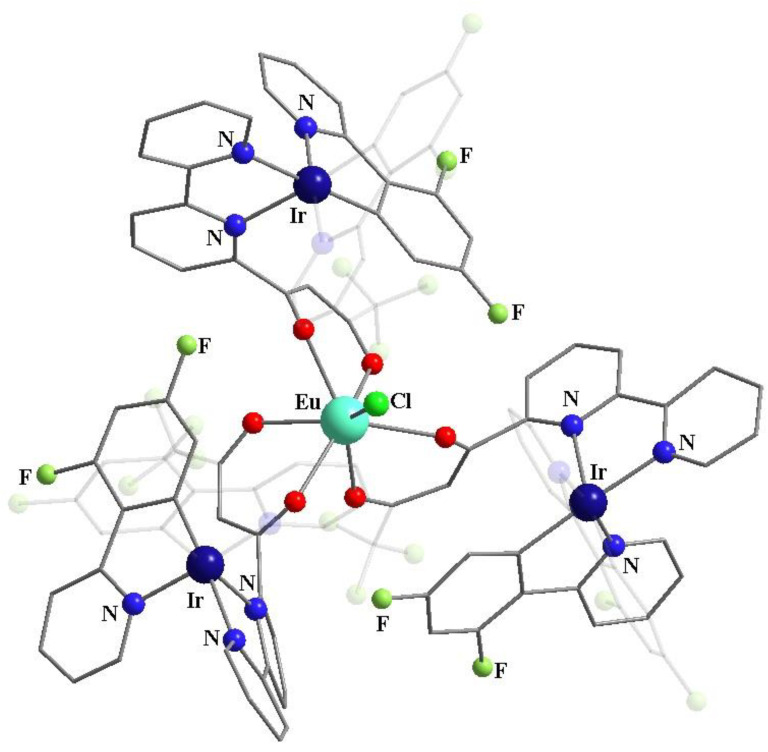
Molecular structure of **50** [52]. Hydrogen atoms have been omitted, some ligand groups are transparent for clarity. The cif has been retrieved from CCDC. CCDC number is 730200.

The fluorinated β-diketonates with 2,2′-bipyridinyl or phenanthrolinyl substituents (**HL^2^**, **HL^3^**) were used in the two-step synthesis of bimetallic [Ir-Eu] complexes **48**, **49** (Scheme 19) [52,53,93,94]. At the first stage, only the 2,2′-bipyridinyl fragment of the ligand coordinated the Ir(III) ion. Furthermore, three β-diketonate anions **48**, **49** with sterically congested iridium(III) fragments were involved in the coordination with lanthanide(III) ions. The resulting compounds **50**, **51** demonstrate the rare examples of seven-coordinated Ln(III) ions with the [LnO_6_Cl] coordination environment (Figure 25). The structural differences of coordination centers promoted the assembly of heteroleptic Ln(III)-Ir(III) (Ln = Eu, Nd, Yb, Er, Gd) complexes [52,53,93,94]. As a result, heterometallic [Ir-Eu] compounds **50**, **51** with bpy fragments demonstrated the higher values of phosphorescence lifetimes (up to 440 μs) compared with phen analogs. Replacing the CF_3_ group with C_2_F_5_ substituent slightly shifted the triplet state in [Ir-Eu] complexes towards the higher side [52]. Therefore, the C_2_F_5_-diketonate modified by bpy moiety represents an example of the efficient europium(III) sensitization through the excitation transferring from the Ir(III) to Eu(III) center [52].

## 9. Metallocene-Containing Heterometallic β-Diketonates

**Figure 26:**
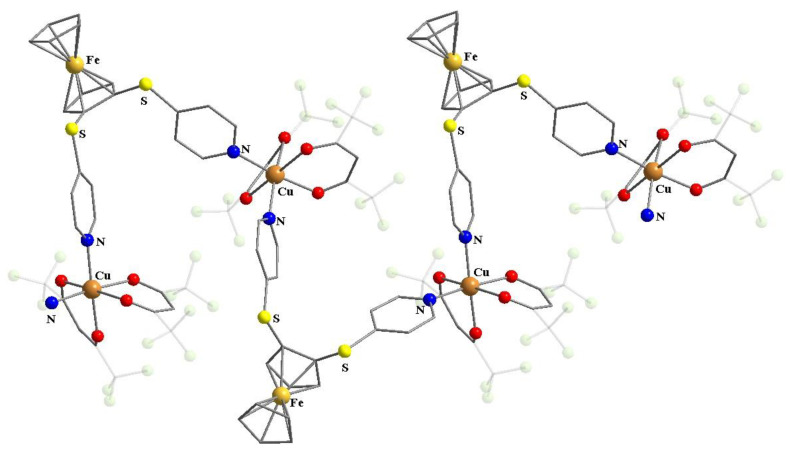
A fragment of polymeric structure of **51** [95]. Hydrogen atoms have been omitted, some ligand groups are transparent for clarity. The cif has been retrieved from CCDC. CCDC number is 1556476.

Metallocene derivatives are convenient molecules for the synthesis of heterometallic compounds with predictable composition and structure. One of the approaches is based on the co-crystallization of the modified ferrocene with transition metal β-diketonates. For example, 1,2-di(4-pyridylthio)ferrocene in reaction with Cu(hfac)_2_ gave 1D coordination polymer [Cu-Fe] (**51**) (Figure 26) [95].

**Scheme 20:**
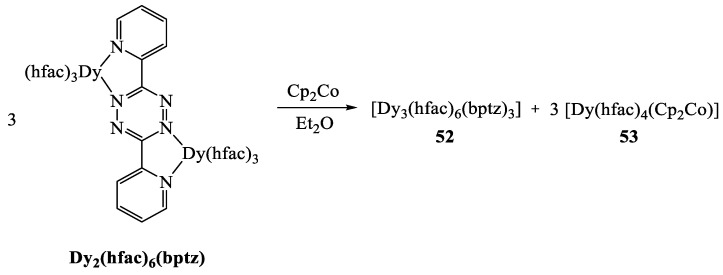
Reaction of Dy_2_(hfac)_6_(bptz) with Cp_2_Co.

**Figure 27:**
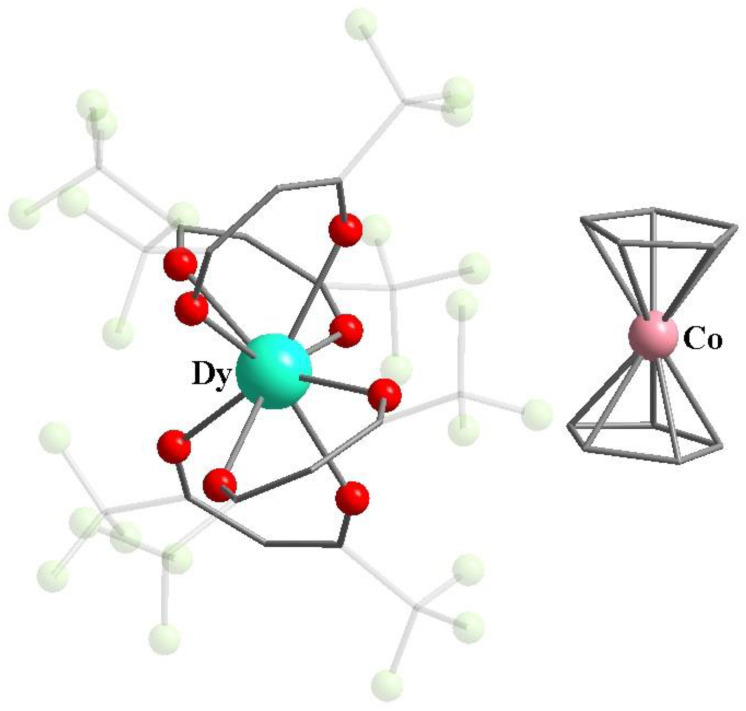
Molecular structure of **53** [96]. Hydrogen atoms have been omitted, some ligands groups are transparent for clarity. The cif has been retrieved from CCDC. CCDC number is 1811780.

Unsubstituted metallocenes can also form the polynuclear structures. Cobalt (I) cyclopentadienyl (Cp_2_Co), acting as a cation in heterometallic Ln(III) *tetrakis*-β-diketonates, afforded a complex **53** with uncoordinated counterions (Figure 27) [96]. The formation of a similar compound as a by-product was observed during the reaction between a binuclear lanthanide complex [Ln_2_(hfac)_6_(bptz)] (**52**) (where bptz–3,6-bis(2-pyridyl)-1,2,4,5-tetrazine) and Cp_2_Co (Scheme 20).

**Scheme 21:**
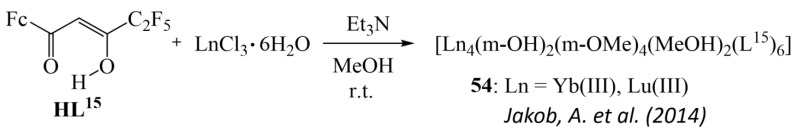
Reaction of β-diketones **HL^15^** with Ln(III) chloride [98].

**Figure 28:**
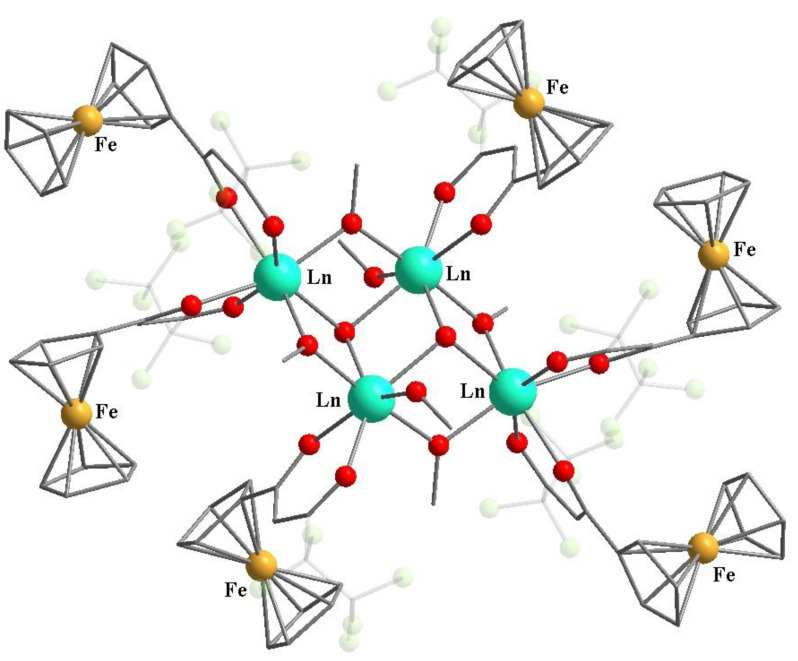
Molecular structure of **54** [97]. Hydrogen atoms have been omitted, C_2_F_5_ groups are transparent for clarity. The cif has been retrieved from CCDC. CCDC number is 984498.

The β-diketones **HL^15^** reacted with Ln(III) chloride in the presence of triethylamine in methanol at room temperature to yield the clusters **54** containing four 4f metal ions decorated with ferrocene rings (Scheme 21) [97]. The organic base promoted the deprotonation of methanol and water molecules followed by the formation of a tetranuclear lanthanide(III) framework due to methanolate and hydroxide anions, which act as *O*-bridging ligands (Figure 28).

**Scheme 22:**
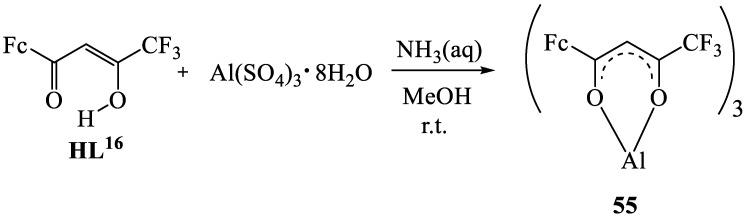
Synthesis of *tris*-β-diketonate [Al-Fe] **55**.

**Figure 29:**
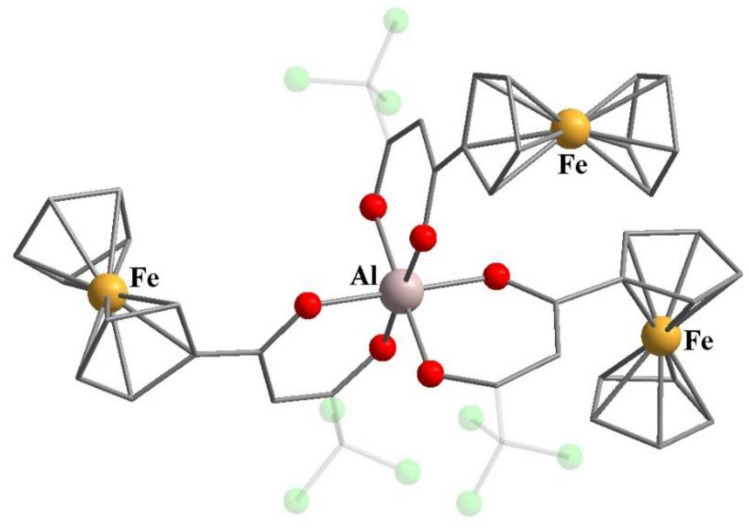
Molecular structure of **55** [99]. Hydrogen atoms have been omitted, CF_3_ groups are transparent for clarity. The cif has been retrieved from CCDC. CCDC number is 877638.

The reaction of diketone **HL^16^** with aluminum(III) sulfate in methanol in the presence of aqueous ammonia resulted in the aluminum *tris*-β-diketonate **55** in 32% yield (Scheme 22) (Figure 29) [99]. The cytotoxicity of complex **55** against the human HeLa neoplastic cells was investigated: it was about 5 times less cytotoxic than the neutral diketone and approximately 50 times less toxic compared with a reference drug cisplatin.

**Scheme 23:**
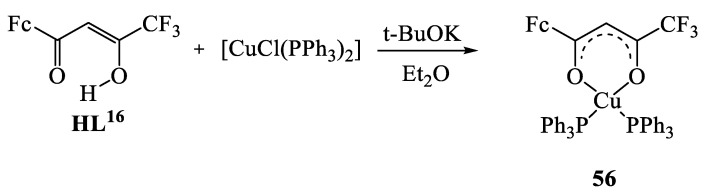
Synthesis of heterometallic [Cu-Fe] complex **56**.

The heterometallic [Cu-Fe] complex **56** was isolated in a good yield (45%) from the reaction of **HL^16^** with [CuCl(PPh_3_)] in the presence of potassium *tert*-butylate in ether at room temperature (Scheme 23) [98]. Complex 56 showed the irreversible oxidation of Cu(I) centers to Cu(II) under anaerobic electrochemical conditions accompanied by the loss of PPh_3_ coligands and the formation of *bis*-diketonate [Cu(**L^16^**)_2_].

**Scheme 24:**
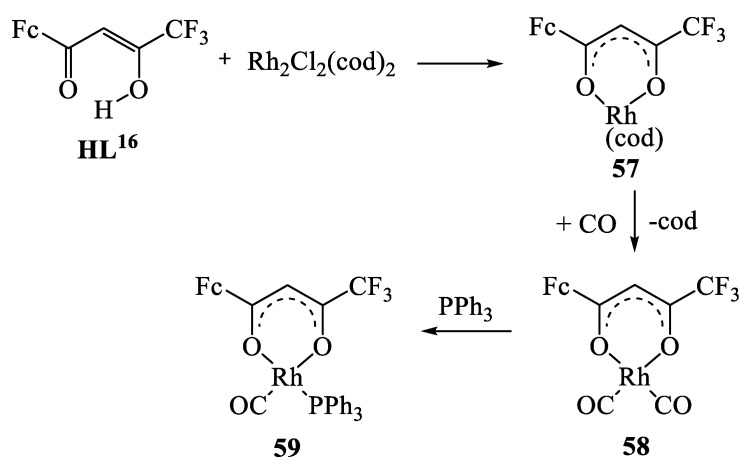
Synthesis of heterometallic [Rh-Fe] complex **59**.

The synthesis of [Rh-Fe] complexes **59** included several stages with the step-by-step replacement of coligands at the Rh(I) ion (Scheme 24) [100]. Compounds **59** have been shown to provide the formation of Rh(I) active centers on the surfaces for heterogeneous catalysis. Silanol groups grafted on a solid matrix primarily replaced the β-diketonate anions in [Rh-Fe] complexes.

**Scheme 25:**
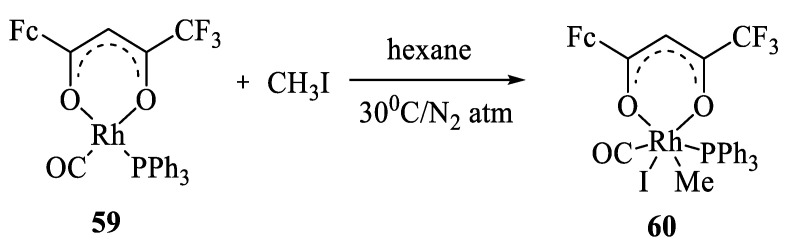
Transformation of heterometallic [Rh-Fe] complex **59**.

**Figure 30:**
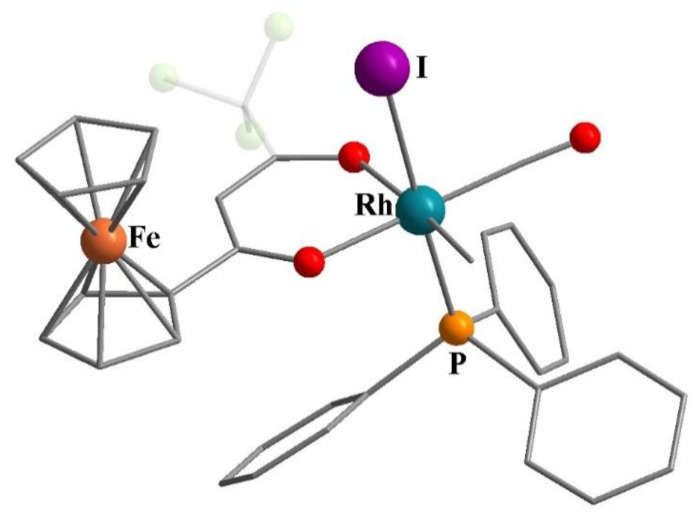
Molecular structure of **60** [101]. Hydrogen atoms have been omitted, CF_3_ group is transparent for clarity. The cif has been retrieved from CCDC. CCDC number is 646438.

The oxidative addition of methyl iodide to the [Rh-Fe] compound **59** proceeded under mild conditions to afford the Rh(III) complex **60** in 90% yield (Scheme 25, Figure 30) [101]. Based on spectral data, the authors postulate that methyl-containing complex **60** and its acyl derivative exist in equilibrium with each other. However, since the ^19^F NMR spectra have not been registered, the transformations of the β-dicarbonyl framework accompanied by the isomers’ formation can proceed as well.

**Scheme 26:**
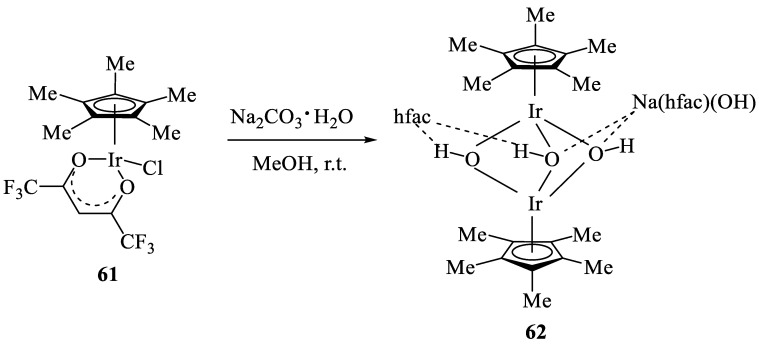
Synthesis of heterometallic [Ir-Na] complex **62**.

**Figure 31:**
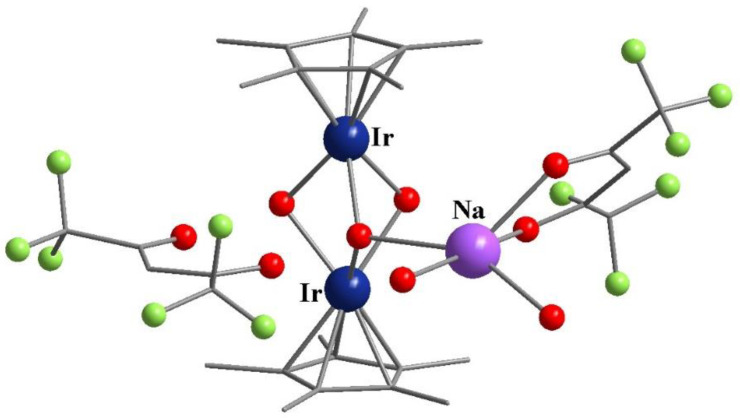
Molecular structure of **62** [102]. Hydrogen atoms have been omitted for clarity. The cif has been retrieved from CCDC. CCDC number is 1996249.

Sodium carbonate reacted with [CpIr(hfac)Cl] (**61**) in methanol under normal conditions to give an unusual heterotrimetallic complex (**62**) (Scheme 26) [102]. This transformation resulted in a hydroxycluster [CpIr(III)] coordinating the fluorinated sodium β-diketonate and free hfac (Figure 31).

## 10. Heteroleptic Complexes Involving β-Diketonates and Polytopic Coligands

**Figure 32:**
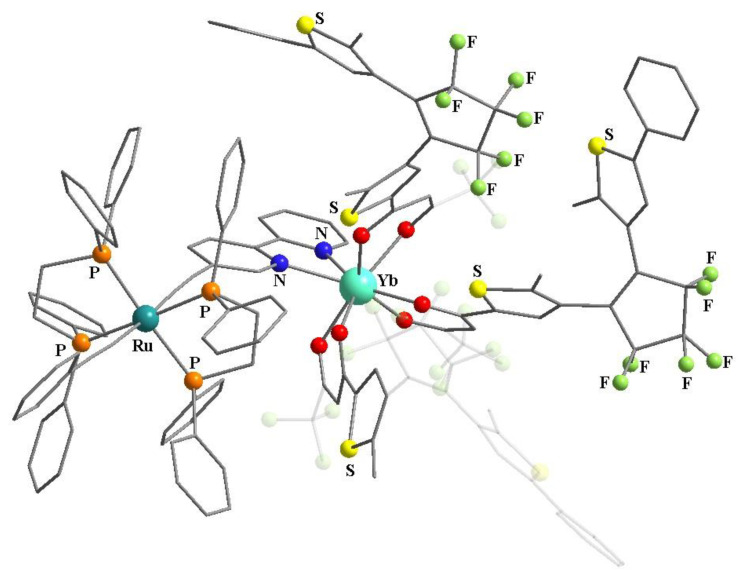
Molecular structure of **63 [51]**. Hydrogen atoms have been omitted, some ligand groups are transparent for clarity. The cif has been retrieved from CCDC. CCDC number is 1959918.

The trifluoromethyl β-diketone **HL^1^** with dithienylethene substituent was used as a ligand in the synthesis of heterometallic [Yb-Ru] complex **63** exhibiting the redox/optical control of emission (Figure 32) [51]. The bimetallic complex **63** was formed due to the coordination of the *tris*-β-diketonate Yb(**L^1^**)_3_ fragment with the functionalized 2,2′-bipyridine containing a ruthenium(III) ion.

**Scheme 27:**
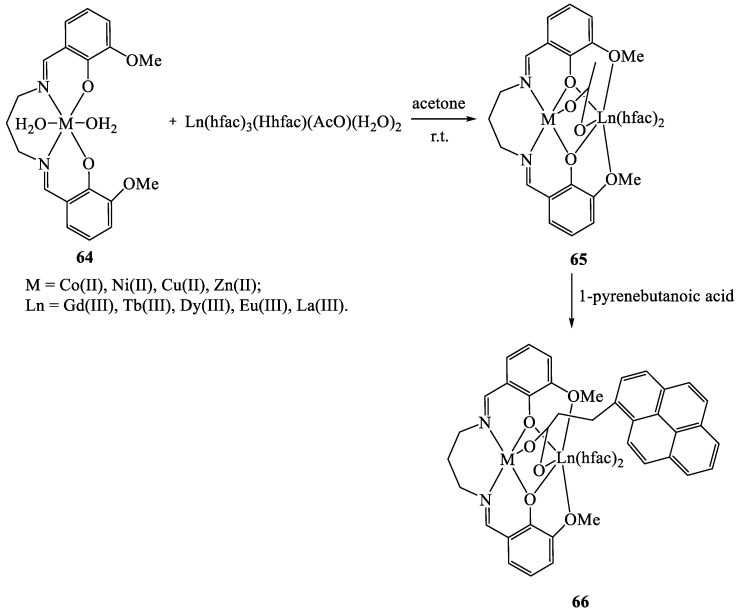
Synthesis of heterometallic 3d-4f binuclear complexes **65–66**.

**Figure 33:**
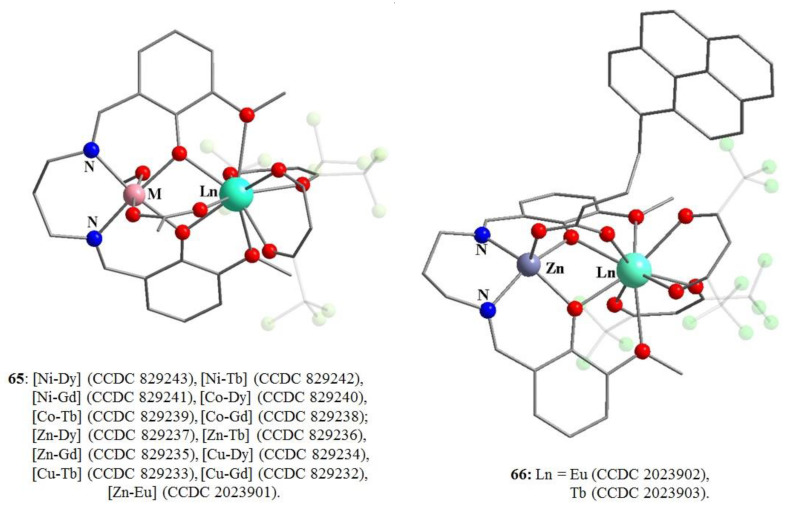
Molecular structure of **65**, **66 (dik** = **hfac)** [103,104]. Hydrogen atoms have been omitted, some ligand groups are transparent for clarity. The cif has been retrieved from CCDC.

The Schiff base with two different coordination modes formed the heterometallic 3d-4f binuclear complexes **65** (Scheme 27) [103,104]. The lanthanide(III) ions had an [LnO_8_] environment, whereas 3d metals ions preferably coordinated with the less “hard” nitrogen centers (Figure 33). The complexes with a combination of anisotropic Co(II) or Ni(II) ions with Tb(III) exhibited slow magnetic relaxation at low temperatures and, therefore, represented promising structures for single-molecular magnets’ design [103]. On the other hand, the heterometallic complexes containing Zn(II) and Tb(III) or Eu(III) ions showed luminescent properties [104]. In this case, the replacement of the bridging acetate anion by 1-pyrenbutanoic acid (complex **66**, Figure 33) reduced the phosphorescence lifetime.

**Figure 34:**
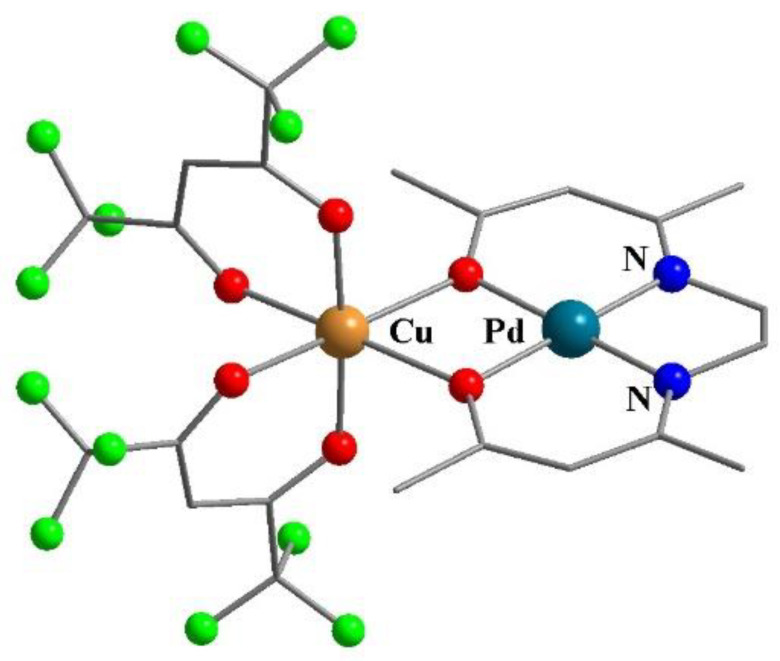
Molecular structure of **67** [105]. The cif has been retrieved from CCDC. CCDC number is 1961116.

The co-crystallization of copper(II) β-diketonate with palladium(II) bis-enaminoketonate led to the heterobimetallic [Cu-Pd] complex **67** [105]. The bridging oxygen atoms of bis-enaminoketone between palladium(II) and copper(II) ions formed a discrete binuclear framework of the complex **67** (Figure 34).

**Scheme 28:**
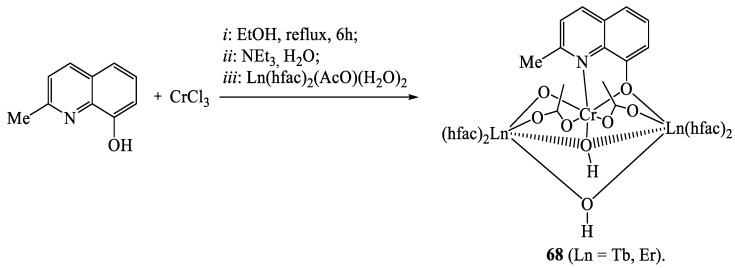
Synthesis of heterometallic [Ln-Cr] complexes **68**.

**Figure 35:**
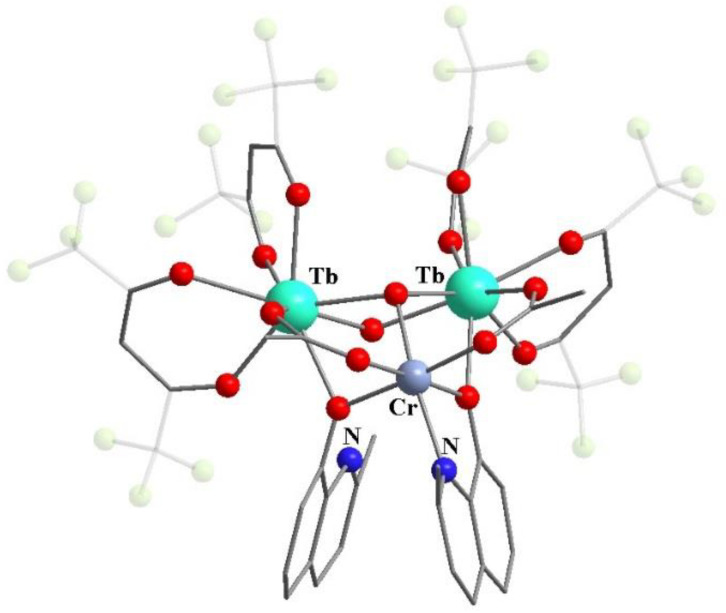
Molecular structure of **68** [108]. CF_3_ groups are transparent for clarity. The cif has been retrieved from CCDC. CCDC number is 812496.

Due to deprotonation of the hydroxy group, 8-hydroxyquinoline acted as a bridging ligand in the reaction with transition metal β-diketonates resulting in the homobimetallic complexes (Scheme 28) [106,107,108]. However, in the presence of a chromium(III) ion a trimetallic complex **68** was formed, in which a bridging phenolate anion connected the different metal ions (Figure 35) [108].

**Scheme 29:**
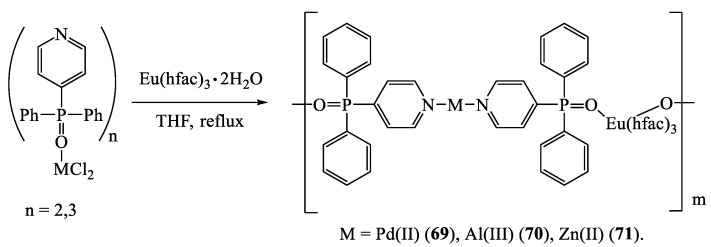
Synthesis of polymers, containing Eu(III) and 3d metal ions.

Heterometallic polymer complexes **69**–**71** including Eu(III) *tris*-β-diketonate fragments were synthesized based on the bidentate *O,N*-ligand–4-pyridyldiphenylphosphinoxide (Scheme 29) [109]. Polymer chains of **69**–**71** were formed through the coordination of phosphinoxide oxygen atom with Eu(hfac)_3_, whereas the nitrogen atom of the pyridine core bound the Pd(II), Zn(II) or Al(III) centers. Among the synthesized luminescent bimetallic polymers, complex [Eu-Al] **70** exhibited the highest value of the phosphorescence quantum yield equal to 72%.

**Figure 36:**
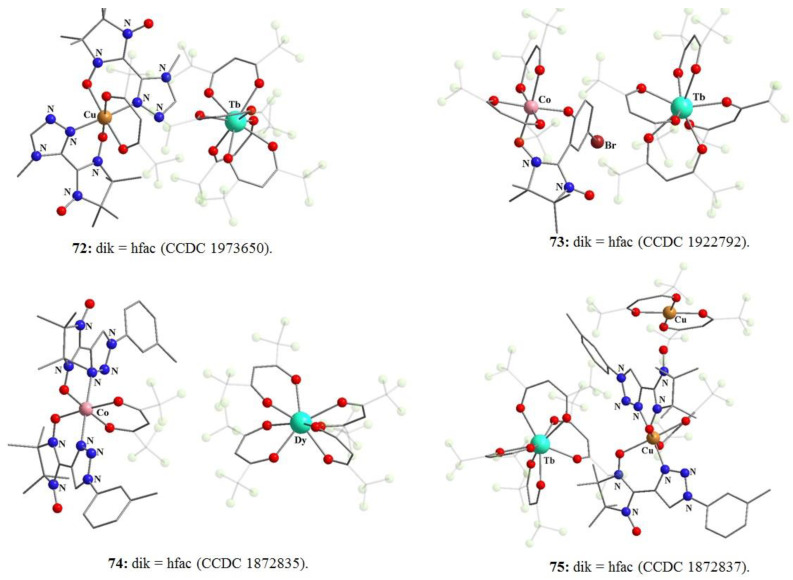
The examples of 3d/4f complexes containing Ln(III) *tetrakis*-β-diketonate fragments. CF_3_ groups are transparent for clarity. The cif has been retrieved from CCDC [110,111,112].

The simultaneous addition of 3d and 4f β-diketonate hydrates to the reaction with nitronyl nitroxyls gave the heterometallic complexes **72**–**75** [110,111,112]. The coordination of 3d β-diketonate with the nitroxyl radical was accompanied by the Ln(III) *tris*-β-diketonate transformation into the corresponding Ln(III) *tetrakis*-β-diketonate anion (Figure 36) [110,111,112].

**Figure 37:**
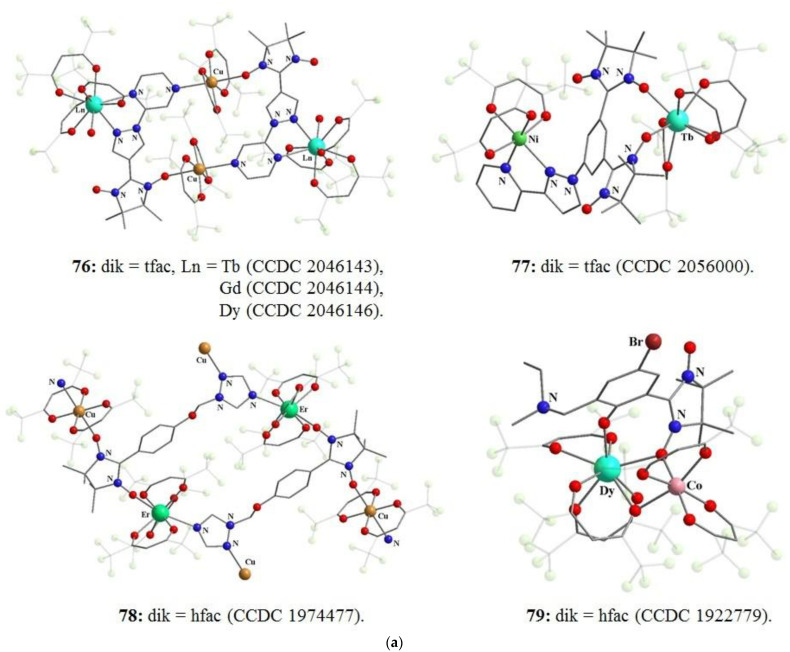

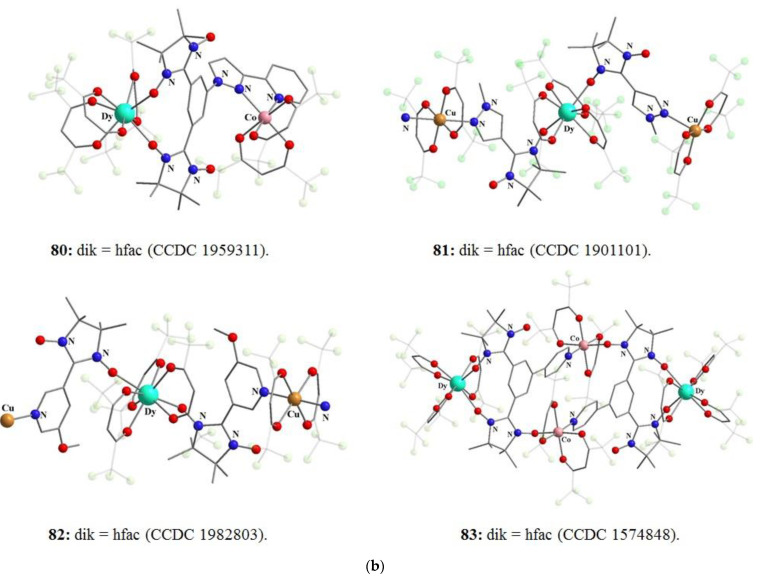
(**a**)The examples of 3d/4f complexes containing Ln(III) *tris*-β-diketonate fragments. CF_3_ groups are transparent for clarity. The cif has been retrieved from CCDC. (**b**) The examples of 3d/4f complexes containing Ln(III) *tris*-β-diketonate fragments. CF_3_ groups are transparent for clarity. The cif has been retrieved from CCDC [113,114,115,116,117,118,119].

In most cases, the co-crystallization of 3d and 4f β-diketonates proceeded in the presence of the aza-heteroaryl-substituted nitronyl nitroxyls to provide the additional coordination centers with metal ions (Figure 37) [113,114,115,116,117,118,119]. The Ln(III) center predominantly formed an [LnO_8_] environment due to both the β-diketonate anions and nitroxyl oxygen atoms. In turn, 3d metal *bis*-diketonates were coordinated by either two nitrogen atoms of the heterocyclic substituents in a ligand or a nitroxyl oxygen atom and aza-heterocycle.

## 11. Heterometallic Complex Synthesis Based on hfac Transformations

In general, the synthesis of mono- and polynuclear β-diketonates is realized under basic conditions to facilitate the coordination with metal ions via deprotonation of the initial β-dicarbonyl compounds. Along with this, in some cases the base action provokes the β-diketone participation in the hydration process, retro-Claisen reaction or template transformations to form the metal complexes including tfa or ttpt as coligands [81,102,120,121,122,123,124,125,126,127,128,129,130].

**Scheme 30:**
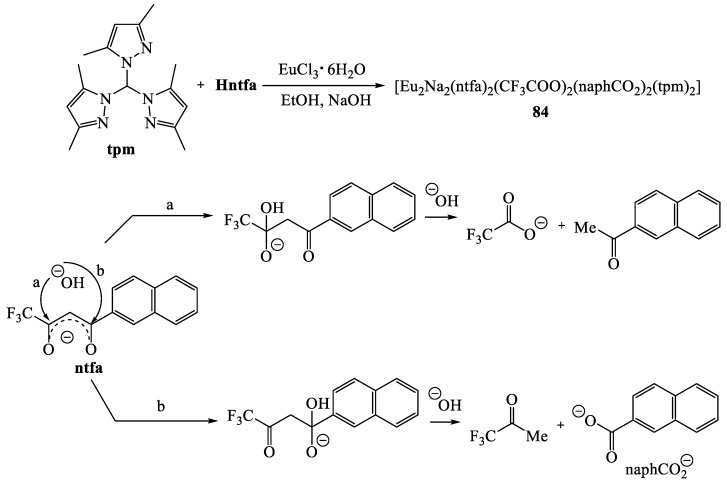
Synthesis and proposed mechanism of heterometallic [Eu-Na] complex (**84**) formation.

**Figure 38:**
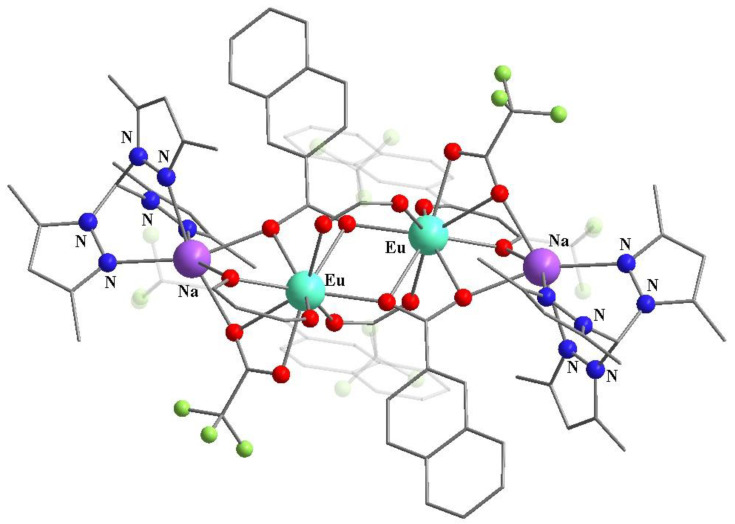
Molecular structure of **84** [126]. Some ligand groups are transparent for clarity. The cif has been retrieved from CCDC. CCDC number is 1008766.

A strong base used in the formation of a β-diketonate anion caused the destruction or transformation of the initial β-diketone. In work [126], the deprotonation with sodium hydroxide resulted in two possible routes of the retro-Claisen reaction (Scheme 30). Surprisingly, in the presence of two different carboxylates, a tetranuclear [Eu-Na] complex (**84**) was formed, including the β-diketonate anions and *tris*(3,5-dimethyl-1-pyrazolyl)methane (tpm) (Figure 38). The main phosphorescence characteristics were determined for the complex **84**: the observed lifetime was 0.68 ms with a quantum yield of 39% and quantum efficiency equal to 58% [126].

**Scheme 31:**
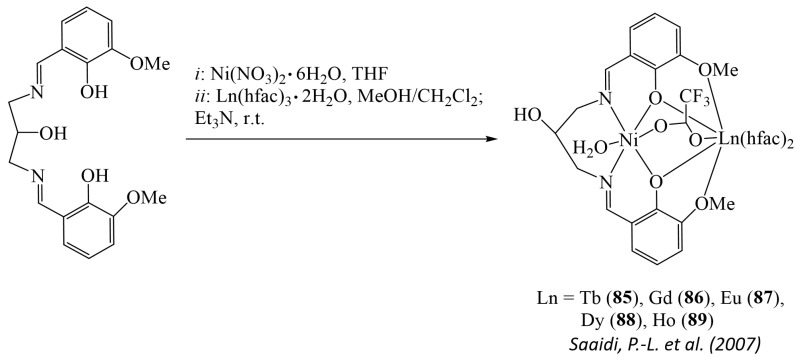
Synthesis of heterometallic [Ln-Ni] complexes **85**–**89** [129].

**Figure 39:**
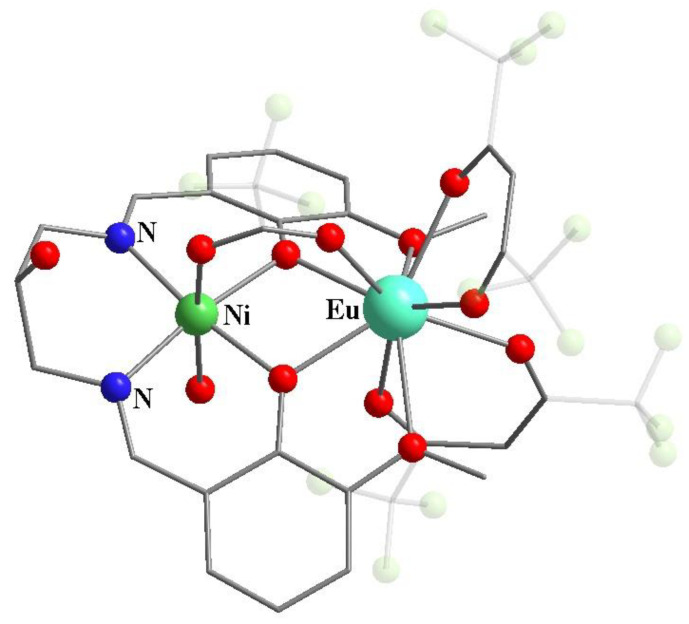
Molecular structure of **87** [127]. CF_3_ groups are transparent for clarity. The cif has been retrieved from CCDC. CCDC number is 1556429.

The synthesis of heterometallic [Ni-Ln] complexes (**83**–**87**) included the two steps: Schiff base interaction with nickel(II) nitrate followed by the Ln(hfac)_3_ addition (Scheme 31) [127]. In that case, one fluorinated β-diketonate molecule from Ln(hfac)_3_ underwent a retro-Claisen reaction to give the [Ni-Ln] complexes (**85**–**89**) with the trifluoroacetate anion as coligand (Figure 39). 

**Scheme 32:**
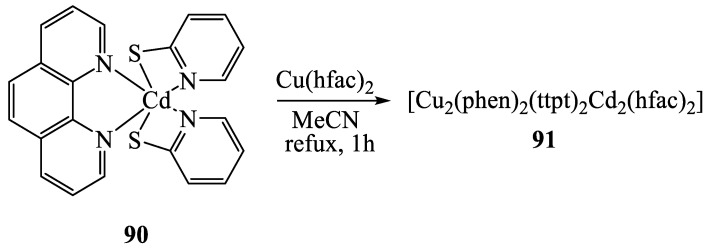
Synthesis of heterometallic [Cu-Cd] complexes **91**.

**Figure 40:**
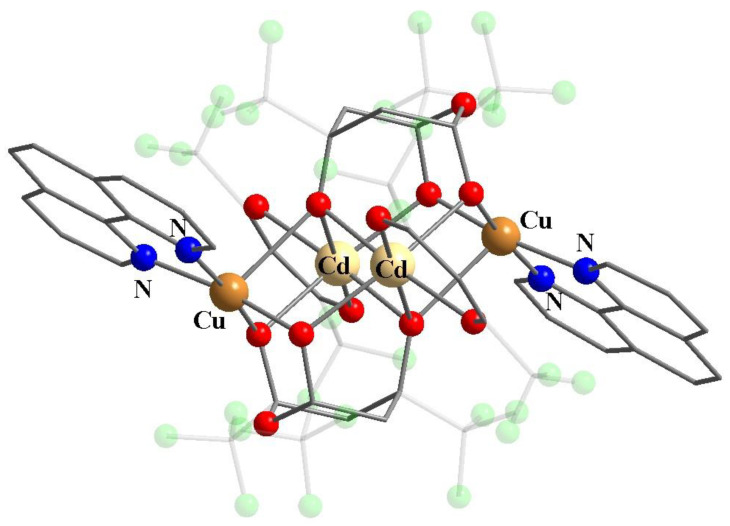
The crystal structures of [Cu_2_(phen)_2_(ttpt)_2_Cd_2_(hfac)_2_] (**91**) [129]. CF_3_ groups are transparent for clarity. The cif has been retrieved from CCDC. CCDC number is 616292.

The nitrogen base, as an initial part of the complex, can further participate in the co-crystallization with fluorinated β-diketonate [129]. In particular, the tetranuclear bimetallic complex **91** was obtained by the reaction of Cd(pymt)_2_(phen)_2_ (**90**) with Cu(hfac)_2_ in acetonitrile (Scheme 32) [129]. The heterometallic structure **91** was formed based on the ttpt derived from hfac. Cadmium(II) both coordinated the diketonate anion and built a heterometallic framework of **91** due to the bridging oxygen atoms of ttpt (Figure 40). In addition, three hydroxyl oxygen atoms of two tpt and phen molecules formed the [CuO_3_N_2_] coordination environment of copper(II) ions.

**Scheme 33:**
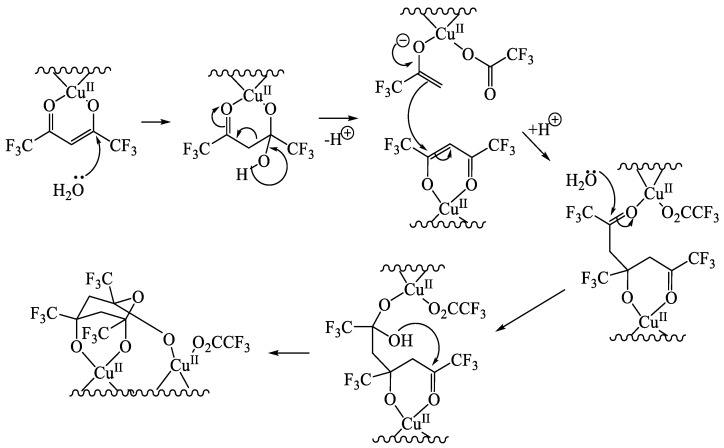
The proposed mechanism of pyran formation from Cu(hfac)_2_.

After the decomplexation of heteronuclear [Cu-Cd] complex **91,** all hydroxyl groups of *tris*-CF_3_-tetrahydropyran-2,4,6-triol were in the *cis*-configuration based on XRD data [129]. The proposed mechanism of pyran formation involves a retro-Claisen reaction followed by a condensation of trifluoroacetone enol with fluorinated copper diketonate (Scheme 33). Obviously, the triol formation led to the assembly of the tetranuclear [Cu-Cd] core, while the oxygen atoms of the hydroxyl groups in ligand acted as bridging atoms between metal ions. The other bases, including DMF, diethylformamide, formamide and sodium hydroxide, can also be used in the template reaction affording the ttpt.

**Scheme 34:**
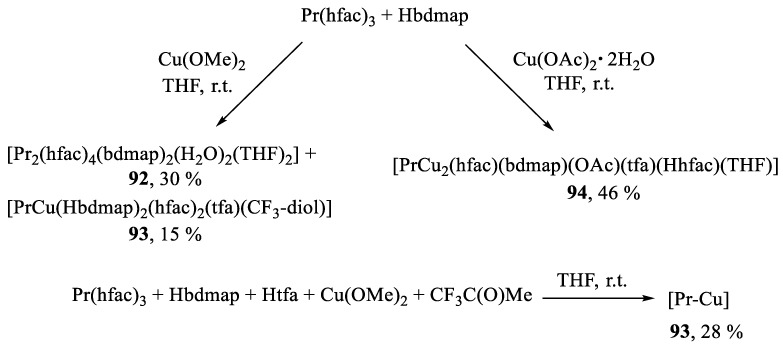
Synthesis of polynuclear Pr(III) complexes via Hhfac transformations.

**Figure 41:**
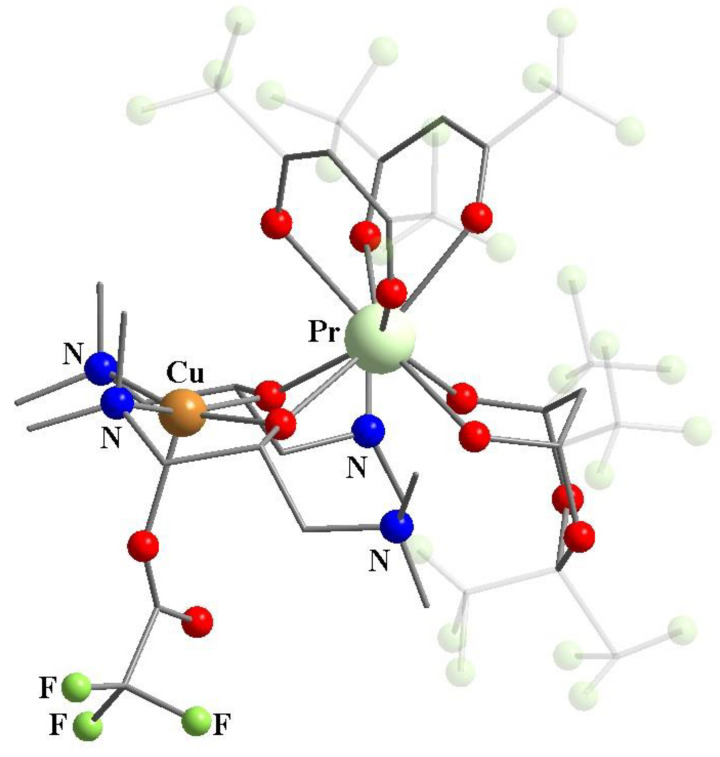
Crystal structure of [Pr(hfac)_2_(CF_3_-diol)Cu(Hbdmap)_2_(tfa)] (**93**) [131]. CF_3_ groups are transparent for clarity. The cif has been retrieved from CCDC. CCDC number is 1232922.

**Scheme 35:**
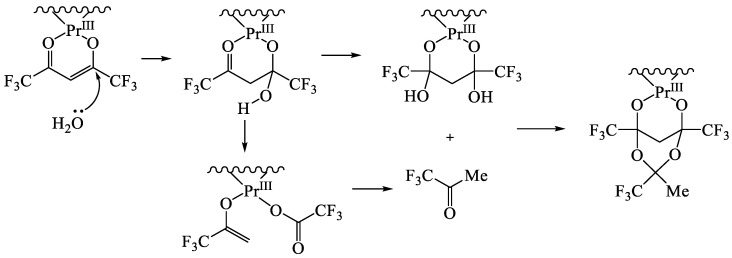
Proposed mechanism for trifluoromethylated dioxane-diol formation.

The Hhfac cleavage accompanied by the trifluoroacetate formation proceeds easily under the action of tertiary amines at room temperature [131]. The reaction between Ln(hfac)_3_ and 1,3-bis(dimethylamino)-2-propanol (bdmap) leads to heteronuclear [Pr-Cu] complexes **93**, **94** (Scheme 34). If copper(II) methoxide is reacted with Pr(hfac)_3_ instead of the corresponding acetate, trifluoroacetate and trifluoromethylated dioxane-diol (CF_3_-diol) are formed as coligands (Figure 41). The CF_3_-diol is formed due to the condensation reaction of trifluoroacetone with β-diketonate from Pr(hfac)_3_ (Scheme 35). To prove the mechanism of heterometallic [Pr-Cu] complex (**93**) formation, the authors carried out the reaction of the copper(II) methoxide and Pr(hfac)_3_ with trifluoroacetic acid and proposed trifluoroacetone in the presence of bdmapH (Scheme 34).

## 12. Conclusions

The described analysis of the literature demonstrates the wide scope of fluorinated β-diketones in the synthesis of heterometallic compounds. In most cases, the available diketones with trifluoromethyl and perfluoropropyl substituents (hfac, fod) form polynuclear systems containing up to five different metal ions. Moreover, fluorinated β-diketones as coligands can be used in the design of the heterometallic architectures in combination with other organic polydentate molecules. However, the potential of coordination compounds containing two or more different metal ions has not been fully realized in terms of practice. This is mainly because of the limited number of transition metals used in the synthesis of heterometallic structures. The specific chemical properties of the initial β-diketones should be considered when planning conditions for the synthesis of coordination compounds. In particular, the action of bases causes the β-diketones’ destruction to form heteroleptic complexes. In this context, the directed synthesis of β-diketones containing additional coordination centers is a more attractive design strategy for the heterometallic complexes [51,52,53,93,94,132]. This area is promising for obtaining not only discrete structures but also 2D/3D organometallic polymers, which are of applied interest in the development of magnetic, fluorescent, catalytic or sensor devices. Therefore, using polydentate fluorinated β-diketonates allows the heterometallic composition of structurally diverse coordination compounds to be varied, which, in turn, may give rise to advanced materials with interesting physicochemical properties.

## Data Availability

Not applicable.

## Abbreviations

MOCVD	metal organic chemical vapor deposition;
hfac	1,1,1,5,5,5-hexafluoro-2,4-pentanedionate;
tfa	trifluoroacetate;
tfac	1,1,1-trifluoro-2,4-pentanedionate;
ptac	1,1,1-trifluoro-5,5-dimethyl-2,4-hexanedionate;
fod	6,6,7,7,8,8,8-heptafluoro-2,2-dimethyl-3,5-octanedionate;
tta	thenoyltrifluoroacetylacetonate;
fta	furanoyltrifluoroacetylacetonate;
bta	benzoyltrifluoroacetone;
ntfa	4,4,4-trifluoro-1-(2-naphtyl)-1,3-butanedionate;
Na_2_EDTA	ethylenediaminetetraacetic acid disodium salt;
PTSA	p-toluenesulfonic acid;
dik	β-diketonate;
acac	acetylacetonate;
thd	2,2,6,6-tetramethyl-3,5-heptanedionate;
pfbm	bis(pentafluorobenzoyl)methane;
Cp	cyclopentadienyl;
Fc	ferrocene;
DME	1,2-dimethoxyethane;
DMF	dimethylformamide;
Et	ethyl;
LDA	lithium di(isopropyl)amide;
Me	methyl;
THF	tetrahydrofuran;
ttpt	*tris*-CF_3_-tetrahydropyran-2,4,6-triol;
bptz	3,6-bis(2-pyridyl)-1,2,4,5-tetrazine;
phen	1,10-phenantroline;
bdmap	1,3-bis(dimethylamino)-2-propanol.
